# An Aqueous Extract of Herbal Medicine ALWPs Enhances Cognitive Performance and Inhibits LPS-Induced Neuroinflammation via FAK/NF-κB Signaling Pathways

**DOI:** 10.3389/fnagi.2018.00269

**Published:** 2018-09-26

**Authors:** Ju-Young Lee, Bitna Joo, Jin Han Nam, Hye Yeon Nam, Wonil Lee, Youngpyo Nam, Yongtaek Seo, Hye-Jin Kang, Hyun-Ji Cho, Young Pyo Jang, Jeongyeon Kim, Young-Man We, Ja Wook Koo, Hyang-Sook Hoe

**Affiliations:** ^1^Department of Neural Development and Disease, Korea Brain Research Institute, Daegu, South Korea; ^2^Department of Brain and Cognitive Sciences, Daegu Gyeongbuk Institute of Science & Technology, Daegu, South Korea; ^3^Division of Pharmacology, College of Pharmacy, Kyung Hee University, Seoul, South Korea; ^4^College of Korean Medicine, Wonkwang University, Iksan, South Korea; ^5^Oriental Medical Clinic Center, Hyoo Medical Clinic, Seoul, South Korea

**Keywords:** LPS, neuroinflammation, NF-κB, IL-1β, FAK

## Abstract

Recent studies have shown that Liuwei Dihuang pills (LWPs) can positively affect learning, memory and neurogenesis. However, the underlying molecular mechanisms are not understood. In the present study, we developed ALWPs, a mixture of *Antler* and LWPs, and investigated whether ALWPs can affect neuroinflammatory responses. We found that ALWPs (500 mg/ml) inhibited lipopolysaccharide (LPS)-induced proinflammatory cytokine IL-1β mRNA levels in BV2 microglial cells but not primary astrocytes. ALWPs significantly reduced LPS-induced cell-surface levels of TLR4 to alter neuroinflammation. An examination of the molecular mechanisms by which ALWPs regulate the LPS-induced proinflammatory response revealed that ALWPs significantly downregulated LPS-induced levels of FAK phosphorylation, suggesting that ALWPs modulate FAK signaling to alter LPS-induced IL-1β levels. In addition, treatment with ALWPs followed by LPS resulted in decreased levels of the transcription factor NF-κB in the nucleus compared with LPS alone. Moreover, ALWPs significantly suppressed LPS-induced BV2 microglial cell migration. To examine whether ALWPs modulate learning and memory *in vivo*, wild-type C57BL/6J mice were orally administered ALWPs (200 mg/kg) or PBS daily for 3 days, intraperitoneally injected (i.p.) with LPS (250 μg/kg) or PBS, and assessed in Y maze and NOR tests. We observed that oral administration of ALWPs to LPS-injected wild-type C57BL/6J mice significantly rescued short- and long-term memory. More importantly, oral administration of ALWPs to LPS-injected wild-type C57BL/6J mice significantly reduced microglial activation in the hippocampus and cortex. Taken together, our results suggest that ALWPs can suppress neuroinflammation-associated cognitive deficits and that ALWPs have potential as a drug for neuroinflammation/neurodegeneration-related diseases, including Alzheimer’s disease (AD).

## Introduction

Microglial cells represent 10–15% of the human brain. Microglia are resident innate immune cells in the central nervous system (CNS) and play a critical role in defending the host in response to exogenous toxins (Kreutzberg, 1996; Hanisch and Kettenmann, 2007; Kettenmann et al., 2011; Tremblay et al., 2011). Upon injury, microglia phagocytize dying cells and any cell debris (Neumann et al., 2009). In addition, microglia maintain brain homeostasis to ensure consistent cytokine levels. In aged brain and rodent models of neurodegenerative disease, microglial activation causes neuroinflammation, synaptic dysfunction and neuronal cell death (Hirsch et al., 2012; Cunningham, 2013; Asai et al., 2015), suggesting an association of neuroinflammation with neurodegenerative diseases. Activated microglial cells significantly increase proinflammatory cytokine levels and Aβ plaque deposition, resulting in impairment of learning and memory (Tan et al., 2013). In Alzheimer’s disease (AD), soluble amyloid-β oligomers and fibrils interact with cell-surface receptors (e.g., integrin, CD14, and the Toll-like receptors TLR2 and TLR4) in microglial cells, and these interactions can also trigger a neuroinflammatory response (Solito and Sastre, 2012; Park et al., 2013; Zhang and Wang, 2014; Heneka et al., 2015). Significantly enhanced microglial activation has been observed in the cortex and hippocampus in mouse models of AD and AD patients, in addition to increased expression of proinflammatory cytokines in the brain, serum, and cerebrospinal fluid (CSF) (Oakley et al., 2006; Dursun et al., 2015; Shal et al., 2018). neuroinflammation has also been implicated in Parkinson’s disease (PD), another neurodegenerative disease. For example, lipopolysaccharide (LPS)-injected wild-type C57BL/6J mice exhibit significantly induced microglial activation, which leads to functional changes such as dopaminergic neuron attenuation in an IL-1-dependent manner, resulting in PD-like behavioral impairment (Tanaka et al., 2013). Interestingly, microglial activation and proinflammatory cytokine levels [e.g., tumor necrosis factor-α (TNF-α), interleukin (IL)-1β, and IL-6] are significantly increased in the brain, CSF, and serum of PD patients postmortem, suggesting potential roles of these markers in predicting the progression of PD (Mogi et al., 1994; Williams-Gray et al., 2016). Although the molecular mechanisms by which neuroinflammation affects neurodegenerative diseases remain to be clarified, modulating microglia-mediated neuroinflammation in the brain may be a therapeutic strategy for treating or preventing neurodegenerative diseases.

Yukmijuhwang-tang (Liu-wei-di-huang-tang in China, Lokumijio-to in Japan) is a traditional Oriental herbal medicine that contains 6 different herb components: steamed *Rehmanniae radix, Discoreae radix, Corni fructus, Hoelen, Moutan Cortex Radicis*, and *Alismatis radix*. Yukmijuhwang-tang is widely used in Asia to treat diabetes mellitus, impaired neurogenesis, and malfunctions of the immune system (Park et al., 2005; Lee et al., 2012). Recent studies have shown that Liuwei Dihuang pills (LWPs), which are prescribed to prevent aging, regulate oxidant and free radical-scavenging activity (Ha et al., 2011; Lee et al., 2012). In addition, LWPs improve learning and memory in aged mice and promote neurogenesis in the dentate gyrus of the hippocampus in a stressed rat model (Wei, 2000; Hsieh et al., 2003; Park et al., 2005; Kang et al., 2006). However, whether LWPs can alter neuroinflammation is unknown.

Deer antler is a traditional herbal medicine that has been reported to restore the immune response, produce anti-inflammatory effects and improve learning and memory. For instance, pilose antler peptide regulates proinflammatory cytokines (e.g., TNF-α and IL-1β) in LPS-induced primary nucleus pulposus cells by inhibiting NF-κB pathways (Dong et al., 2018). Aqueous antler extract (AAE) restores memory impairment in scopolamine (SCOP)-induced memory deficit mice by regulating cholinergic marker enzyme activities (Lee et al., 2010). In addition, secreted molecules from antlers can affect neurite outgrowth and axonal growth (Gray et al., 1992; Li et al., 2007; Pita-Thomas et al., 2010). However, research on the possible roles of antler-derived molecules in inflammation in the brain has been limited.

Based on the literature, we developed ALWPs, which include antler and LWPs (Yukmijuhwang-tang). We anticipated synergistic effects of ALWPs on neuroinflammatory responses and cognitive function. Here, we determined that ALWPs suppressed the LPS-induced neuroinflammatory response in BV2 microglial cells but not primary astrocytes, suggesting that ALWPs can differentially affect neuroinflammatory responses depending on cell type. In addition, ALWPs altered TLR4/FAK/NF-κB signaling to regulate LPS-induced neuroinflammatory responses. Moreover, oral administration of ALWPs to LPS-injected wild-type C57BL/6J mice resulted in rescue of short- and long-term memory and significantly reduced microglial activation. Taken together, these data suggest that ALWPs have potential as an anti-inflammatory drug, including for AD.

## Materials and Methods

### Ethics Statement

All experiments were approved by the Institutional Biosafety Committee (IBC) of the Korea Brain Research Institute (approval no. 2014-479).

### Animals

All experiments were performed in accordance with the approved animal protocols and guidelines established by the Korea Brain Research Institute (IACUC-2016-0013). C57BL6/J mice were purchased from Orient-Bio Company (Gyeonggi-do, South Korea). Male wild-type C57BL6/J mice (8 weeks of age, 25–30 g) were housed in a pathogen-free facility with 12 h of light and dark per day at an ambient temperature of 22°C. Water and food were available *ad libitum*. Mice were housed in groups of 3–5 per cage, *randomly* assigning *animals* to control/treatment *groups* for all experiments. Cotton nestlets were provided to minimize stress effects. Data were analyzed in a semi-automated manner using ImageJ software, and the results were confirmed by an independent researcher who did not participate in the current experiments.

### LPS Neuroinflammation Model

Lipopolysaccharide was injected intraperitoneally (i.p.) to evoke neuroinflammation in wild-type C57BL/6J mice as previously described (Lee et al., 2008). The animals were divided into three experimental groups for each experiment: group 1 was treated with phosphate-buffered saline (PBS); group 2 was treated with both LPS and PBS; and group 3 was treated with both LPS and ALWPs (200 mg/kg). To test the effects of ALWPs on cognitive function, 8- to 9-week-old wild-type mice were orally administered ALWPs (200 mg/kg, p.o.) or PBS daily for 3 days. On the third day, 1 h after administration of PBS or ALWPs, LPS was injected i.p. at a dose of 250 μg/kg. The injected dose of LPS was selected based on a previous study (Fruhauf et al., 2015). One hour after LPS injection, the Y-maze test and a training session for the novel object recognition test (NOR) were performed sequentially. The NOR test was conducted 24 h after the NOR training session. To examine the effects of ALWPs on cognitive performance, we used 29 mice for the Y-maze test and 24 mice for the NOR test. In the NOR test, mice that had less than 7 s of exploration time during training and test were excluded from analysis (Taglialatela et al., 2009; Wolf et al., 2016).

### Y-Maze Test

The Y-maze test was performed to assess immediate working memory as described previously (Fruhauf et al., 2015). The mice were placed in a Y-shaped maze with 3 arms at angles of 120° from each other (36.5 cm × 7 cm × 15.5 cm) under low red light (4–5 lux). During a 3-min trial, the mice freely moved and explored the arms. The number of arms visited and their sequences were recorded, and alternation triplets were analyzed manually after video recording. The percentage of alternation (%) was calculated using the following formula: number of alternation triples/(total number of arm entries-2) × 100.

### Novel Object Recognition (NOR)

The NOR task was performed to assess long-term memory as described previously (Fruhauf et al., 2015). On the training day, mice were placed in an experimental apparatus (30 cm × 30 cm × 25 cm) containing two identical objects under low red light (4–5 lux). The mice moved freely to explore the objects for 3 min. The next day, the mice were placed in the same apparatus with two objects for 5 min for the test sessions. One item was a familiar object, that is, the same object experienced previously, whereas the other was a novel object that the mouse had never experienced before. All trials were videotaped, and the interaction times were measured manually. The times during a mouse passed near or climbed above the object were not counted; only the time during which the mouse was heading to the object was measured. The relative interaction time was calculated as the preference (%) based on the following formula: T_Novel_/(T_Familiar_ + T_Novel_) × 100, where T is the time of interaction with an object.

### Antibodies and Chemicals

We used the following primary antibodies: mouse anti-β-actin (Cat No: sc-47778, Santa Cruz Biotechnology, Dallas, TX, United States), mouse anti-p-IκBα (B-9, Cat No: sc-8404, Santa Cruz Biotechnology), mouse anti-IκBα (Cat No: sc-1643, Santa Cruz Biotechnology), rabbit anti-NF-κB (P65, Cat No: sc-8008, Santa Cruz Biotechnology), mouse anti-PCNA (Cat No: sc-56, Santa Cruz Biotechnology), rat anti-mouse CD11b (M1/70, Cat No: ab8878, Abcam, Cambridge, MA, United States), rabbit anti-FAK (Cat No: 13009S, Cell Signaling Technology, Danvers, MA, United States), rabbit anti-p-FAK (Tyr397, Cat No: 8556S, Cell Signaling Technology), rabbit anti-p-NF-κB (Ser536, Cat No: 3033L, Cell Signaling Technology), rabbit anti-ERK (Cat No: 9102S, Cell Signaling Technology), rabbit anti-p-ERK (Thr202/Tyr204, Cat No:9101S, Cell Signaling Technology), rabbit anti-JNK (Cat No: MBS8509129, MyBioSource, San Diego, CA, United States), rabbit anti-p-JNK (Thr183/Tyr185, Cat No: MBS8508944, MyBioSource), rabbit anti-P38 (Cat No: 9212S, Cell Signaling Technology), rabbit anti-p-P38 (Cat No: 9211S, Cell Signaling Technology), rabbit anti-TLR4 (Cat No: PA5-11597, Thermo Scientific, Waltham, MA, United States), and rabbit anti-TLR4 (Cat No: NB100-56566, Novus Biologicals, Littleton, CO, United States). Horseradish peroxidase (HRP)-conjugated anti-mouse (Cat No: ADI-SAB-100-J, Enzo Life Science, NY, United States), and rabbit IgG (Cat No: ADI-SAB-300-J, Enzo Life Science, NY, United States) were used as the secondary antibodies. LPS from *Escherichia coli* O111:B4 (Cat No: L2880, Sigma, St. Louis, MO, United States) and the TLR4 inhibitor TAK-242 were purchased from Calbiochem (Cat No: 614316, San Diego, CA, United States), and the p-FAK inhibitor PF-573228 (Cat No: S2031) was purchased from Selleckchem (Houston, TX, United States).

### Cell Lines and Culture Conditions

BV2 cells were derived from primary mouse microglial cells. BV2 microglial cells (a gift from Dr. Kyoungho Suk at Kyungpook National University) were maintained in high-glucose DMEM (HyClone, Logan, UT, United States) supplemented with 5% fetal bovine serum (FBS, HyClone) in a 5% CO_2_ incubator. Treatments of BV2 microglial cells were always performed in serum-free DMEM. All BV2 microglial cells used in *in vitro* experiments were validated by immunostaining with anti-CD11b antibody, as a microglial cell marker. We also used BV2 microglia cells within 10 passages to ensure that healthy cells were used in the experiments. Data from all *in vitro* experiments were analyzed in a semi-automated manner using ImageJ software, and the results were confirmed by an independent researcher who did not participate in the current experiments.

### Primary Astrocyte Cultures

Primary astrocytes were cultured from the cerebral cortices of 1-day-old Sprague Dawley rats. Briefly, the cortices were triturated into single cells in high-glucose DMEM containing 10% FBS and penicillin-streptomycin solution (5000 units/ml penicillin, 5 μg/ml streptomycin; Corning, NY, United States) and plated on 75-cm^2^ T flasks (0.5 hemisphere/flask) for 2 weeks. To prepare astrocyte-enriched cultures, the microglia were detached by shaking for 2 h at 120 rpm, and the cells remaining in the flask were harvested with 0.1% trypsin. The astrocytes were plated in 12-well culture plates (35-mm dish) pre-coated with poly-D-lysine (Sigma-Aldrich).

### Cell-Surface Biotinylation

BV2 microglial cells were treated with ALWPs (500 μg/ml) or PBS for 30 min followed by LPS (1 μg/ml) or PBS for 5.5 h. Surface proteins were then labeled with Sulfo-NHS-SS-Biotin under gentle shaking at 4°C (Cat No: 89881, Thermo Scientific, United States). After 30 min, quenching solution was added to the cells. The surface-labeled cells were lysed in lysis buffer, disrupted by sonication on ice, incubated for 30 min, and clarified by centrifugation (10,000 × *g*, 10 min). After centrifugation, the lysate was added to immobilized NeutrAvidin^TM^ gel and incubated for 1 h. After washing three times with wash buffer, the samples were incubated for 1 h in SDS–PAGE sample buffer with DTT. Surface proteins were then analyzed by immunoblotting with an antibody recognizing the N-terminus of TLR4.

### Cytosol and Nuclear Fractionation

BV2 microglial cells were lysed in cytosol fractionation buffer (10 mM HEPES pH 8.0, 1.5 mM MgCl_2_, 10 mM KCl, 0.5 mM DTT, 300 mM sucrose, 0.1% NP-40, and 0.5 mM PMSF) and centrifuged for 5 min at 10,000 rpm. The supernatant was collected as the cytosolic fraction. The remaining pellet was resuspended in nuclear fractionation buffer (10 mM HEPES pH 8.0, 20% glycerol, 100 mM KCl, 100 mM NaCl, 0.2 mM EDTA, 0.5 mM DTT, and 0.5 mM PMSF), incubated on ice for 15 min, and centrifuged for 1 min at 10,000 rpm. The supernatant was collected as the nuclear fraction (Nam et al., 2017).

### Reverse Transcription PCR (RT-PCR)

To examine the effects of ALWPs on IL-1β, IL-6, COX-2, iNOS, and TNF-α mRNA levels, BV2 or primary astrocyte cells were pretreated with PBS or ALWPs (250 or 500 μg/ml) for 30 min and then treated with PBS or LPS (1 μg/ml) for 5.5 h. RNA was then extracted using TRIzol (Ambion, United States). RT-PCR was performed with the following primers for BV2 cells: IL-1β: Forward (F)′, AGC TGG AGA GTG TGG ATC CC and Reverse (R)′, CCT GTC TTG GCC GAG GAC TA; IL-6: F′, CCA CTT CAC AAG TCG GAG GC and R′, GGA GAG CAT TGG AAA TTG GGG T; IL-18: F′, TTT CTG GAC TCC TGC CTG CT and R′, ATC GCA GCC ATT GTT CCT GG; COX-2: F′, GCC AGC AAA GCC TAG AGC AA and R′, GCC TTC TGC AGT CCA GGT TC; iNOS: F′, CCG GCA AAC CCA AGG TCT AC and R′, GCA TTT CGC TGT CTC CCC AA; TNF-α: F′, CTA TGG CCC AGA CCC TCA CA and R′, TCT TGA CGG CAG AGA GGA GG; GAPDH: F′, CAG GAG CGA GAC CCC ACT AA and R′, ATC ACG CCA CAG CTT TCC AG. For primary astrocytes, the following primers were used for RT-PCR:COX-2: F′, TCC AAC TCA AGT TCG ACC CA and R′, TCC TCC GAA GGT GCT AGG TT; IL-1β: F′, AAA ATG CCT CGT GCT GTC TG and R′, CAG AAT GTG CCA CGG TTT TC; IL-6: F′, TTG CCT TCT TGG GAC TGA TG and R′, TGG AAG TTG GGG TAG GAA GG; iNOS: F′, ATC ATG GAC CAC CAC ACA GC and R′, GGT GTT GAA GGC GTA GCT GA; TNF-α: F′, AGC ACA GAA AGC ATG ATC CG and R′, CTC CCT CAG GGG TGT CCT TA; GAPDH: F′, GTT ACC AGG GCT GCC TTC TC and R′, GTG ATG GCA TGG ACT GTG GT. Image analyses were performed using ImageJ software to measure the average band intensities.

### Preparation of ALWPs

ALWPs included LWPs (Yukmijuhwang-tang: *Rehmannia glutinosa, Cornus officinalis, Dioscoreae rhizoma, Paeonia suffruticosa, Poria cocos*, and *Alisma orientale*), *Lycium chinense, Polygala tenuifolia, Acorus gramineus*, and *Antler*. The ALWP formula was prepared via a three-step extraction process. First, *L. chinense* (1000 g), *R. glutinosa* (1200 g), *D. rhizoma* (500 g), *C. officinalis* (500 g), *P. suffruticosa* (360 g), *A. orientale* (360 g), *P. cocos* (360 g), and *Antler* (240 g) were boiled for 12 h at 120°C. Second, the heated mixture was sifted through a filter to remove debris. *P. tenuifolia* (180 g), *A. gramineus* (180 g), and *P. cocos* (90 g) were added to the reaction mixture from the first step and boiled for 1 h to prepare a cohesive agent. Finally, the cohesive agent was air-dried for 72 h and molded into a pill. The pills were stored at 4°C, and the extract was dissolved in PBS and diluted with medium before each experiment. To verify each herbal component of ALWPs and to determine the content of the principal markers, ultra-high-performance liquid chromatography (UHPLC) was performed. In addition, to examine the effects of the individual components of ALWPs on LPS-induced proinflammatory cytokine levels, we calculated the doses of the individual components of the mixture from the composition formula of ALWPs and conducted further experiments.

### Liquid Chromatography

Reverse-phase UHPLC was performed on a Waters ACQUITYUHPLC H-Class system (Milford, MA, United States) consisting of a quaternary solvent manager pump, sample manager – TN and photodiode array (PDA) detector. Empower 3 Pro software (Milford, MA, United States) was used for UHPLC data analysis. Chromatographic separation was accomplished on a Perkin Elmer C18 reverse-phase column (Brownlee SPP 100 mm × 1.0 mm I.D., 1.7 μm) at 30°C and monitored at 283 nm for 5-HMF (5-hydroxymethyl-2-furaldehyde, Sigma Aldrich), 231 nm for paeoniflorin (MFDS, Osong, South Korea), 240 nm for morroniside (ChemFaces, Wuhan, China), and 237 nm for loganin (MFDS). A solvent system consisting of acetonitrile (solvent A) and 0.1% formic acid DW (solvent B) was used with a gradient from 2% (solvent A): 98% (solvent B) to 100% (solvent A): 0% (solvent B) over 18 min at a flow rate of 0.4 ml/min. Standard stock solutions were prepared by dissolving standards in 100% methanol to obtain a final concentration of 10 μg/ml. After ultracentrifugation at 9000 rpm and filtration through a syringe filter (0.2 μg, Whatman^TM^, Maidstone, United Kingdom), 4 ml of sample was injected six times.

### MTT Assays

Cell viability was assessed using the 3-(4,5-dimethylthiazol-2-yl)-2,5-diphenyltetrazolium bromide (MTT) assay. Cells were seeded in 96-well plates and treated with various concentrations of ALWPs (50–250 μg/ml) for 6 or 24 h in the absence of FBS. The cells were then treated with 0.5 μg/ml MTT and incubated for 3 h at 37°C in a 5% CO_2_ incubator. After discarding the culture medium, dimethyl sulfoxide (DMSO) was added to dissolve the formazan dye, and the absorbance was measured at 580 nm.

### Immunocytochemistry

Cells were plated at a density of 20,000 cells/well on cover slips in 12-well plates (Eppendorf, Hamburg, Germany). Twenty four hour after exchanging the culture medium with serum-free high-glucose DMEM (HyClone), the cells were pretreated with ALWPs (250 μg/ml) or PBS for 30 min, followed by treatment with LPS (1 μg/ml) or PBS for 5.5 h. The cells were then fixed in cold methanol for 8 min, washed with PBS three times, and incubated with one of the following primary antibodies in GDB buffer [0.1% gelatin, 0.3% Triton X-100, 16 mM sodium phosphate (pH 7.4), and 450 mM NaCl] overnight at 4°C: anti-CD11b (M1/70, Cat No: ab8878, 1:200, Abcam), anti-IL-1β (Cat No: sc-7884, 1:200, Santa Cruz Biotechnology), or p-NF-κB (Ser536, Cat No: 3033L, 1:100, Cell Signaling Technology). The next day, the cells were washed with PBS three times and incubated with the following secondary antibodies for 1 h at room temperature: Alexa Fluor 488 and Alexa Fluor 555 (1:200, Molecular Probes, OR, United States). The cells were mounted in DAPI-containing solution (Vector Laboratories, CA, United States), and images were acquired on a single plane using a confocal microscope (Nikon, Japan) and analyzed using ImageJ software.

### Immunofluorescence Analysis

To examine the effects of ALWPs on microglial activation, 3-month-old wild-type C57BL/6J mice were orally administered ALWPs (200 mg/kg, p.o.) or PBS daily for 3 days. On the third day, LPS (10 mg/kg) was injected i.p. Three hours later, the mice were perfused and fixed in 4% paraformaldehyde (PFA) solution, and brain tissues were flash-frozen and dissected using a cryostat (35 μm thick). Each brain section was processed for immunofluorescence staining. The sections were rinsed with PBS and incubated with rabbit anti-Iba-1 (1:500, Wako, Japan) to detect microglia. Primary antibodies were diluted with 0.5% bovine serum albumin (BSA) and incubated at 4°C overnight. The following day, the tissues were rinsed with 0.5% BSA and incubated with Alexa Fluor 555-conjugated anti-rabbit IgG (1:500, Molecular Probes) for 1 h at room temperature. The tissues were subsequently mounted on gelatin-coated cover glass and covered with DAPI-containing mounting solution (Vector Laboratories). The stained tissues were imaged using confocal microscopy (TI-RCP, Nikon). Nine mice were used for immunofluorescence analysis.

### Western Blotting

Cells were lysed using IPH lysis buffer (50 mM Tris-Cl, 150 mM NaCl, 1 % NP-40, 0.5 M EDTA, 1 mM PMSF, 0.1 M DTT) containing protease and phosphatase inhibitor tablets (Roche, Basel, Switzerland). Equal amounts of protein (10 or 20 μg) were mixed with sample loading buffer (Bio-Rad, Hercules, CA, United States), boiled for 5 min, and separated by SDS–PAGE using a Mini protein Tetra cell system. The separated proteins were transferred to polyvinylidene difluoride membranes (PVDF, Millipore, Temecula, CA, United States) using an electrophoretic transfer system (Bio-Rad). After blocking with 5% non-fat dry milk or 5% BSA for 1 h at room temperature, the membranes were incubated with specific primary antibodies as follows: mouse β-actin (1:5000), mouse anti-p-IκBα (1:1000), rabbit anti-IκBα (1:1000), rabbit anti-NF-κB (P65, 1:1000), mouse anti-PCNA (1:1000), rabbit anti-FAK (1:1000), rabbit anti-p-FAK (Tyr397, 1:1000), rabbit anti-ERK (1:1000), rabbit anti-p-ERK (Thr202/Tyr204, 1:1000), rabbit anti-JNK (1:1000), rabbit anti-p-JNK (Thr183/Tyr185, 1:1000), rabbit anti-P38 (1:1000), rabbit anti-p-P38 (1:1000), rabbit anti-TLR4 (1:1000), and rabbit anti-TLR4 (1:1000) or secondary antibodies (HRP-conjugated anti-rabbit or mouse, 1:10,000). Finally, the membrane were developed with enhanced chemiluminescence detection reagents (ATTO, Japan) (Song et al., 2016). Images were analyzed using Fusion software or ImageJ software.

### Cell Migration Assay (Wound-Healing Assay)

The wound-healing assay was performed as previously described (Nam et al., 2017). BV2 microglial cells were seeded in 12-well plates and incubated until the cells reached 80–90% confluence. The cells were then scratched with a cell scratcher (SPL, Gyeonggi-do, South Korea) to create a wound. Images were captured immediately or after 24 h.

### Statistical Analyses

All data were analyzed using either unpaired two-tailed *t*-tests with Welch’s correction for comparisons between two groups or one-way ANOVA for multiple comparisons in GraphPad Prism 6 software. *Post hoc* analyses were completed with Tukey’s multiple comparison test with significance set at ^∗^*p* < 0.05, ^∗∗^*p* < 0.01, ^∗∗∗^*p* < 0.0001. Data are presented as the mean ± SEM.

## Results

### Effects of ALWPs on Cell Viability and Morphology in BV2 Microglial Cells

**FIGURE 1:**
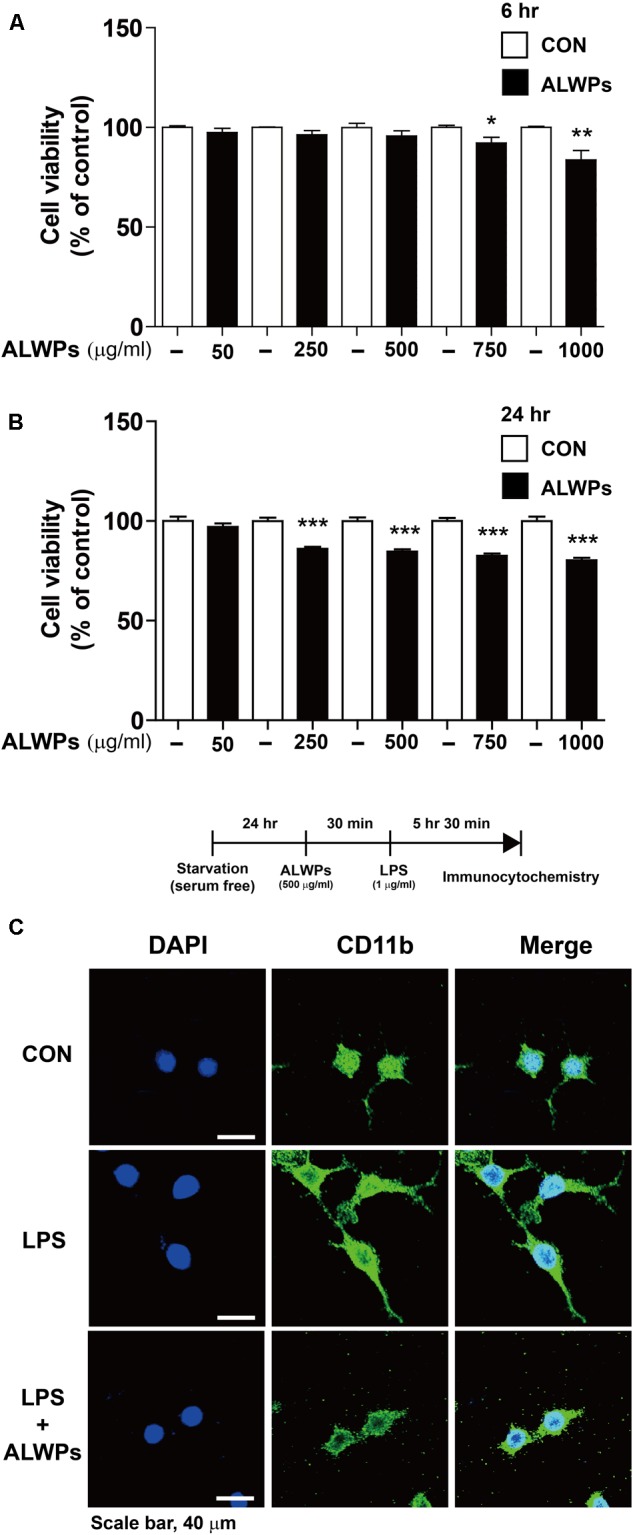
The effects of ALWPs on cell viability and morphology in BV2 microglial cells. **(A,B)** BV2 microglial cells were treated with PBS or ALWPs (50, 250, 500, 750, and 1000 μg/ml) at various concentrations for 6 h [**(A)**, *n* = 8 for each dose] or 24 h [**(B)**, *n* = 16 for each dose], and cell viability was measured. **(C)** Morphological changes of LPS-induced and ALWP-treated BV2 microglial cells, scale bar 40 μm (40× confocal images). ^∗^*p* < 0.05, ^∗∗^*p* < 0.01, ^∗∗∗^*p*< 0.001.

To assess the effects of ALWPs on cell viability, we treated BV2 microglial cells with PBS or ALWPs (50, 250, 500, 750, or 1000 μg/ml) for 6 h and then conducted MTT assays. ALWPs did not cause cell toxicity at concentrations up to 500 μg/ml, but cell viability was reduced when the ALWPs concentration was 750 μg/ml or higher (**Figure 1A**).

Next, we examined whether ALWPs affected cell viability under longer treatment times. BV2 microglial cells were treated with PBS or ALWPs (50, 250, 500, 750, or 1000 μg/ml) for 24 h, and MTT assays were conducted. ALWPs slightly decreased BV2 microglial cell viability at the highest doses, with reductions of 17.5 and 19.8% at 750 and 1000 μg/ml, respectively (**Figure 1B**). Based on these findings, we selected 250 and 500 μg/ml as optimal ALWP concentrations for the subsequent experiments.

To examine whether ALWPs affect cell morphology, we treated BV2 microglial cells with either PBS or ALWPs (500 μg/ml) for 30 min followed by LPS (1 μg/ml) or PBS for 5.5 h. The cells were then fixed and immunostained with an anti-CD11b antibody as a marker for microglia. Compared with the control, LPS-treated cells displayed long, thin, fiber-like structures extending from the cell body (**Figure 1C**, middle panel), consistent with previous studies (Sheng et al., 2011; Dang et al., 2014). However, cells treated with ALWPs followed by LPS exhibited shorter branches and a rounder cell body shape compared with cells treated with LPS (**Figure 1C**, lower panel).

### ALWPs Significantly Decrease LPS-Mediated IL-1β Levels in BV2 Microglial Cells

**FIGURE 2:**
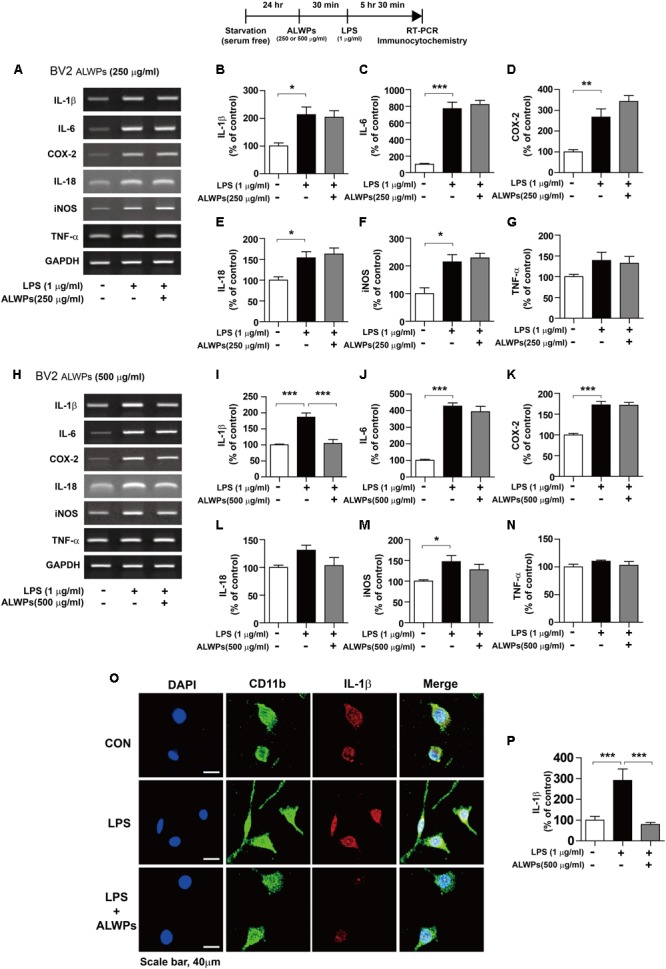
ALWPs inhibit LPS-induced IL-1β levels in BV2 microglial cells. **(A–G)** BV2 microglial cells were pretreated with ALWPs (250 μg/ml) or PBS for 30 min, followed by treatment with LPS (1 μg/ml) or PBS for 5.5 h. Total RNA was isolated, and the mRNA levels of proinflammatory cytokines were measured by RT-PCR (con, *n* = 4; LPS, *n* = 4; ALWPs + LPS, *n* = 4). **(H–N)** BV2 microglial cells were pretreated with ALWPs (500 μg/ml) or PBS for 30 min, followed by treatment with LPS (1 μg/ml) or PBS for 5.5 h. Total RNA was isolated, and the mRNA levels of proinflammatory cytokines were measured by RT-PCR (IL-1β, IL-6, IL-18, iNOS, and TNF-α: con, *n* = 4; LPS, *n* = 4; ALWPs + LPS; *n* = 4, COX-2: con, *n* = 8; LPS, *n* = 8; ALWPs + LPS, *n* = 8). **(O)** BV2 microglial cells were pretreated with ALWPs (500 μg/ml) or PBS for 30 min, followed by treatment with LPS (1 μg/ml) or PBS for 5.5 h. Cells were fixed and immunostained with anti-CD11b and anti-IL-1β antibodies. **(P)** Quantification of the data from **(O)** (con, *n* = 70 cells; LPS, *n* = 80 cells; LPS + ALWPs, *n* = 82, 40× confocal images). ^∗^*p* < 0.05, ^∗∗^*p* < 0.01, ^∗∗∗^*p* < 0.001.

Several studies have shown that the components of ALWPs, specifically *R. glutinosa* and *Corni fructus*, can regulate inflammation in several organs (Park et al., 2005; Lee et al., 2012). However, whether the components of ALWPs can alter neuroinflammation has not been determined. Thus, we examined whether ALWPs can regulate LPS-mediated proinflammatory cytokine levels. BV2 microglial cells were pretreated with ALWPs (250 μg/ml) or PBS for 30 min, followed by treatment with LPS (1 μg/ml) or PBS for 5.5 h. Total RNA was isolated, and the mRNA levels of proinflammatory cytokines were measured by RT-PCR. Surprisingly, 250 μg/ml ALWPs did not alter any LPS-induced proinflammatory cytokine levels in BV2 microglial cells (**Figures 2A–G**).

We then examined whether higher concentrations of ALWPs can alter LPS-induced proinflammatory responses. BV2 microglial cells were pretreated with ALWPs (500 μg/ml) or PBS for 30 min, followed by treatment with either LPS (1 μg/ml) or PBS for 5.5 h. Total RNA was isolated, and the mRNA levels of proinflammatory cytokines were examined by RT-PCR. Interestingly, pretreatment with ALWPs followed by LPS significantly reduced the mRNA levels of IL-1β in LPS-stimulated BV2 microglial cells but not the mRNA levels of other proinflammatory cytokines (**Figures 2H–N**).

To further confirm the findings described above, BV2 microglial cells were pretreated with ALWPs (500 μg/ml) or PBS for 30 min, followed by treatment with either LPS (1 μg/ml) or PBS for 5.5 h. Immunostaining was performed with anti-CD11b and anti-IL-1β antibodies. Consistent with the findings described above, LPS alone significantly increased IL-1β levels compared with the control treatment, but pretreatment with ALWPs followed by LPS treatment significantly decreased IL-1β levels compared with LPS treatment (**Figures 2O,P**). These results suggest that ALWPs can selectively affect LPS-induced proinflammatory cytokine levels in BV2 microglial cells.

**FIGURE 3:**
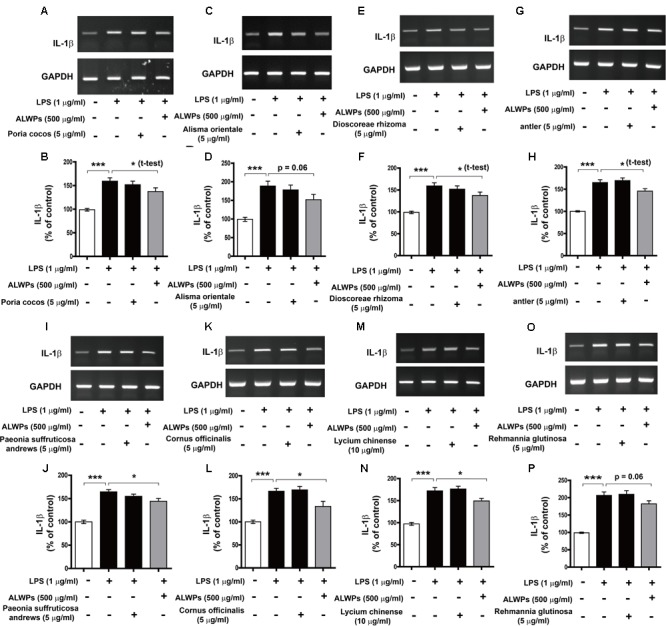
The effects of each component of ALWPs on LPS-induced IL-1β mRNA levels. **(A,B)** BV2 microglial cells were pretreated with *Poria cocos* (5 μg/ml), ALWPs (500 μg/ml), or PBS for 30 min, followed by treatment with LPS (1 μg/ml) or PBS for 5.5 h. **(C,D)** BV2 microglial cells were pretreated with *Alisma orientale* (5 μg/ml), ALWPs (500 μg/ml), or PBS for 30 min, followed by treatment with LPS (1 μg/ml) or PBS for 5.5 h. **(E,F)** BV2 microglial cells were pretreated with *Dioscoreae rhizoma* (5 μg/ml), ALWPs (500 μg/ml), or PBS for 30 min, followed by treatment with LPS (1 μg/ml) or PBS for 5.5 h. **(G,H)** BV2 microglial cells were pretreated with *Antler* (5 μg/ml), ALWPs (500 μg/ml), or PBS for 30 min, followed by treatment with LPS (1 μg/ml) or PBS for 5.5 h. **(I,J)** BV2 microglial cells were pretreated with *Paeonia suffruticosa Andrews* (5 μg/ml), ALWPs (500 μg/ml), or PBS for 30 min, followed by treatment with LPS (1 μg/ml) or PBS for 5.5 h. **(K,L)** BV2 microglial cells were pretreated with *Cornus officinalis* (5 μg/ml), ALWPs (500 μg/ml), or PBS for 30 min, followed by treatment with LPS (1 μg/ml) or PBS for 5.5 h. **(M,N)** BV2 microglial cells were pretreated with *Lycium chinense* (10 μg/ml), ALWPs (500 μg/ml), or PBS for 30 min, followed by treatment with LPS (1 μg/ml) or PBS for 5.5 h. **(O,P)** BV2 microglial cells were pretreated with *Rehmannia glutinosa* (5 μg/ml), ALWPs (500 μg/ml), or PBS for 30 min, followed by treatment with LPS (1 μg/ml) or PBS for 5.5 h. After treatment, total RNA was isolated, and the mRNA levels of proinflammatory cytokines were measured by RT-PCR **(B,D,F,H,J,L,N,P)**. Quantification of the data from **(A)** (vehicle, *n* = 31; LPS, *n* = 31; *P. cocos* + LPS, *n* = 31; ALWPs + LPS, *n* = 31), **(C)** (vehicle, *n* = 13; LPS, *n* = 13; *A. orientale* + LPS, *n* = 13; ALWPs + LPS, *n* = 13), **(E)** (vehicle, *n* = 9; LPS, *n* = 9; *D. rhizoma* + LPS, *n* = 9; ALWPs + LPS, *n* = 9), **(G)** (vehicle, *n* = 12; LPS, *n* = 12; *Antler* + LPS, *n* = 12; ALWPs + LPS, *n* = 12), **(I)** (vehicle, *n* = 12; LPS, *n* = 12; *P. suffruticosa Andrews* + LPS, *n* = 12; ALWPs + LPS, *n* = 12), **(K)** (vehicle, *n* = 9; LPS, *n* = 9; *C. officinalis* + LPS, *n* = 9; ALWPs + LPS, *n* = 9), **(M)** (vehicle, *n* = 17; LPS, *n* = 17; *L. chinense* + LPS, *n* = 17; ALWPs + LPS, *n* = 17), and **(O)** (vehicle, *n* = 41; LPS, *n* = 41; *R. glutinosa* + LPS, *n* = 41; ALWPs + LPS, *n* = 41). ^∗^*p* < 0.05, ^∗∗∗^*p* < 0.001.

To determine whether the individual components of ALWPs can alter proinflammatory cytokine IL-1β levels compared with treatment with ALWPs and LPS or with LPS alone, BV2 microglial cells were pretreated with *P. cocos* or ALWPs (500 μg/ml) for 30 min, followed by treatment with either LPS (1 μg/ml) or PBS for 5.5 h. Total RNA was isolated, and the mRNA levels of IL-1β were examined by RT-PCR. The same methodology was used to treat BV2 microglial cells with *A. orientale, D. rhizoma, Antler, P. suffruticosa Andrews, C. officinalis, L. chinense*, or *R. glutinosa*. Consistent with the findings described above, ALWPs blocked LPS-induced proinflammatory cytokine IL-1β mRNA levels compared with LPS (**Figures 3A–P**, ANOVA with *post hoc* Tukey’s test, two-tailed *t*-test of LPS vs. ALWPs + LPS). In addition, we observed that pretreatment with the individual components of ALWPs followed by LPS treatment did not significantly alter IL-1β mRNA levels compared with treatment with LPS alone (**Figures 3A–P**). These data suggest that the combination of these ten components in ALWPs may have additive/synergistic effects on the neuroinflammatory response.

### ALWPs Do Not Alter LPS-Induced Proinflammatory Cytokine Levels in Primary Astrocytes

To examine whether ALWPs can regulate proinflammatory cytokine levels in primary astrocytes, primary astrocytes were first treated with either ALWPs (500 μg/ml) or PBS for 30 min and then treated with LPS (1 μg/ml) or PBS for 5.5, 11.5, or 23.5 h. After the specified treatment period, total RNA was isolated from the cells, and proinflammatory cytokine mRNA levels were measured by RT-PCR. We found that ALWP treatment did not alter LPS-mediated proinflammatory cytokine levels at any time point in primary astrocytes (**Supplementary Figure 1**).

We then examined whether ALWPs affect proinflammatory cytokine levels in a dose-dependent manner. Primary astrocytes were treated with either ALWPs (500, 750, and 1000 μg/ml) or PBS for 30 min and then treated with LPS (1 μg/ml) or PBS for 5.5 h. The cells were harvested to isolate total RNA, and proinflammatory cytokine levels were measured by RT-PCR. Again, we found that no dosage of ALWPs regulated LPS-mediated proinflammatory cytokine levels in primary astrocytes (**Supplementary Figure 2**). These data suggest that ALWPs may differentially regulate LPS-induced proinflammatory responses in specific cell types.

### ALWPs Decrease LPS-Induced Cell-Surface TLR4 in BV2 Microglial Cells

**FIGURE 4:**
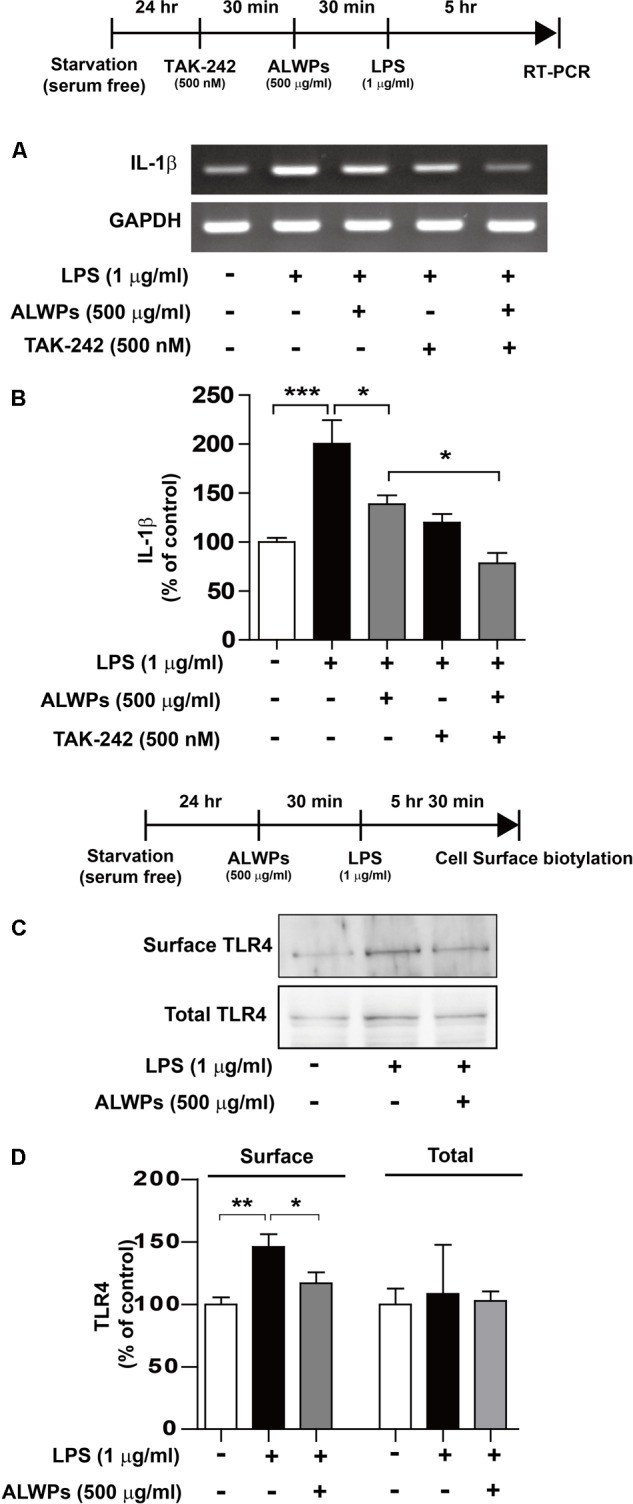
ALWPs significantly reduced LPS-induced cell-surface levels of TLR4. **(A)** BV2 microglial cells were pretreated with TAK-242 (a TLR4 inhibitor, 500 nM) for 30 min, followed by treatment with ALWPs (500 μg/ml) or PBS for 30 min and finally LPS (1 μg/ml) or PBS for 5 h. Total RNA was isolated, and IL-1β mRNA levels were measured by RT-PCR. **(B)** Quantification of the data from **(A)** (con, *n* = 20; LPS, *n* = 20; ALWPs + LPS, *n* = 20; TAK-242 + LPS, *n* = 20; TAK-242 + ALWPs + LPS, *n* = 20). **(C)** BV2 microglial cells were pretreated with ALWPs (500 μg/ml) or PBS for 30 min, followed by treatment with LPS (1 μg/ml) or PBS for 5.5 h. Cell-surface biotinylation was then conducted with a TLR4 antibody recognizing the N-terminal region of TLR4. **(D)** Quantification of the data from **(C)** (Surface TLR4: con, *n* = 10; LPS, *n* = 10; ALWPs + LPS, *n* = 10; Total TLR4: con, *n* = 4; LPS, *n* = 4; ALWPs + LPS, *n* = 4). ^∗^*p* < 0.05, ^∗∗^*p* < 0.01, ^∗∗∗^*p* < 0.001.

Several studies have implied that LPS can activate microglial cells by interacting with Toll-like receptors (e.g., TLR4) (Chen et al., 2012; Hines et al., 2013; Yao et al., 2013b; Dai et al., 2015). Therefore, we examined whether TLR4 can alter LPS-induced IL-1β mRNA levels in the presence of ALWPs. BV2 microglial cells were treated with TAK-242 (a TLR4 inhibitor, 500 nM) for 30 min, ALWPs (500 μg/ml) or PBS for 30 min, and finally LPS (1 μg/ml) or PBS for 5 h. Total RNA was then isolated, and IL-1β mRNA levels were measured by RT-PCR. Consistent with the results shown in **Figure 3**, ALWP treatment followed by LPS decreased IL-1β mRNA levels compared with LPS alone (**Figures 4A,B**). In addition, treatment with TAK-242, ALWPs, and LPS did not reduce IL-1β mRNA levels compared with treatment with TAK-242 and LPS (**Figures 4A,B**). These data suggest that ALWPs alter TLR4 to affect LPS-induced proinflammatory cytokine levels.

Next, we examined whether ALWPs can modulate cell-surface levels of TLR4. BV2 microglial cells were first treated with ALWPs (500 μg/ml) or PBS for 30 min and then treated with LPS (1 μg/ml) or PBS for 5.5 h. Cell-surface biotinylation was performed with a TLR4 antibody recognizing the N-terminus of TLR4. LPS treatment significantly increased the cell-surface levels of TLR4 (**Figures 4C,D**). In addition, treatment with ALWPs followed by LPS significantly reduced the cell-surface levels of TLR4 compared with LPS alone (**Figures 4C,D**). These results suggest that ALWPs inhibit cell-surface levels of TLR4 and thereby decrease the interaction between LPS and TLR4 on the cell surface to regulate proinflammatory responses.

### ALWPs Alter FAK Signaling to Modulate LPS-Induced IL-1β mRNA Levels

To investigate the molecular mechanisms by which ALWPs modulate LPS-stimulated proinflammatory responses, we initially tested whether ALWPs modulate MAP kinase signaling cascades, a downstream LPS-induced signaling pathway (Kaminska et al., 2009; Dang et al., 2014; Guo et al., 2016). BV2 microglial cells were treated with ALWPs (500 μg/ml) or PBS for 5 h and then treated with LPS (1 μg/ml) or PBS for 45 min, and western blotting was performed with anti-p-ERK and anti-ERK antibodies. ALWPs did not alter p-ERK or total ERK levels in LPS-stimulated BV2 microglial cells (**Supplementary Figures 3A,B**). We then examined whether ALWPs can regulate other MAP kinase signaling pathways, including P38 and JNK signaling. Again, ALWPs did not alter the phosphorylation status and total level of P38/JNK in LPS-induced BV2 microglial cells (**Supplementary Figures 3C–F**).

**FIGURE 5:**
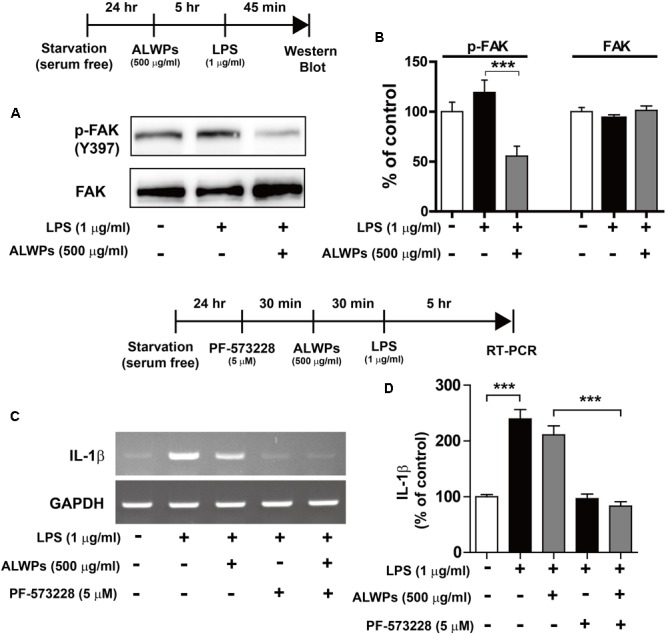
ALWPs decreased LPS-induced FAK phosphorylation in BV2 microglial cells. **(A)** BV2 microglial cells were pretreated with ALWPs (500 μg/ml) or PBS for 5 h, followed by treatment with LPS (1 μg/ml) or PBS for 45 min, and western blotting was performed with anti-p-FAK and anti-FAK antibodies. **(B)** Quantification of the data from **(A)** (p-FAK and FAK: con, *n* = 8; LPS, *n* = 8; ALWPs + LPS, *n* = 8). **(C)** BV2 microglial cells were pretreated with PF-573228 (a FAK inhibitor, 5 μM) for 30 min, followed by treatment with ALWPs (500 μg/ml) or PBS for 30 min and finally LPS (1 μg/ml) or PBS for 5 h. Total RNA was isolated, and IL-1β mRNA levels were measured by RT-PCR. **(D)** Quantification of the data from **(C)** (con, *n* = 30; LPS, *n* = 30; ALWPs + LPS, *n* = 30; PF-573228 + LPS, *n* = 30; PF-573228 + ALWPs + LPS, *n* = 30). ^∗∗∗^*p* < 0.001.

Next, we examined whether ALWPs can regulate the phosphorylation of focal adhesion kinase (FAK) as a potential target involved in inflammation (Zeisel et al., 2005; Wong et al., 2011; Choi et al., 2015; Lian et al., 2015). BV2 microglial cells were pretreated with ALWPs (500 μg/ml) or PBS for 5 h and then treated with LPS (1 μg/ml) or PBS for 45 min, and western blotting was performed with anti-p-FAK and anti-FAK antibodies. Cells treated with LPS alone showed an increase in the phosphorylation of FAK compared with the control treatment (**Figures 5A,B**), and ALWPs significantly decreased LPS-induced FAK phosphorylation compared with LPS treatment in BV2 microglial cells (**Figures 5A,B**).

To investigate whether ALWPs alter FAK signaling to affect LPS-induced IL-1β mRNA levels, BV2 microglial cells were treated with PF-573228 (a FAK inhibitor, 5 μM) or vehicle (1% DMSO) for 30 min, ALWPs (500 μg/ml) or PBS for 30 min, and finally LPS (1 μg/ml) or PBS for 5 h. Total RNA was isolated, and IL-1β mRNA levels were measured by RT-PCR. Treatment with PF-573228, ALWPs, and LPS did not reduce LPS-induced IL-1β mRNA levels compared with treatment with PF-573228 and LPS (**Figures 5C,D**). These results suggest that ALWPs regulate FAK signaling to modulate LPS-stimulated proinflammatory responses.

### ALWPs Suppress LPS-Induced NF-κB Levels in the Nucleus

**FIGURE 6:**
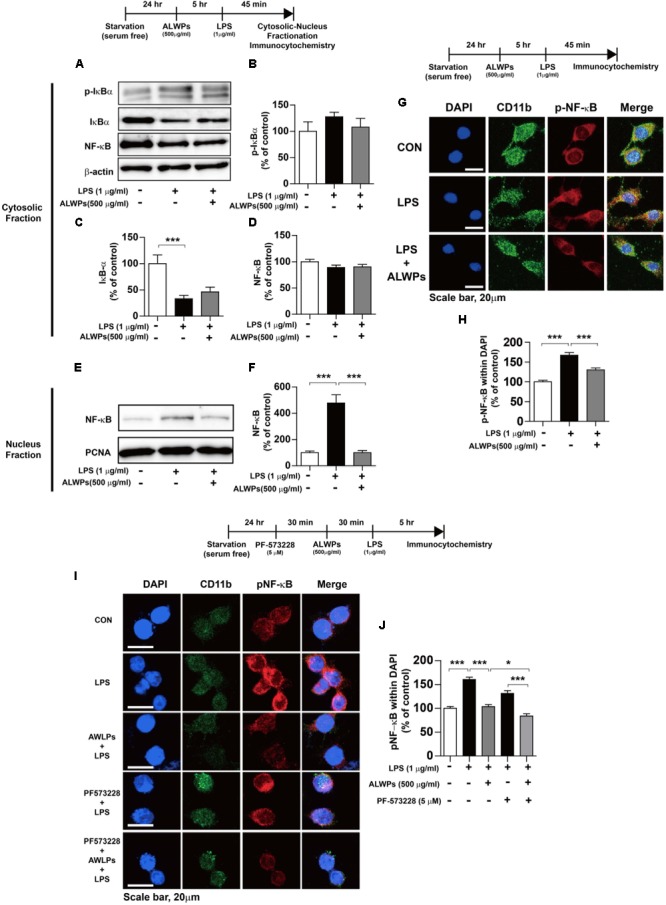
ALWPs decreased LPS-induced nuclear NF-κB (Ser536) levels. **(A)** BV2 microglial cells were pretreated with ALWPs (500 μg/ml) or PBS for 5 h, followed by treatment with LPS (1 μg/ml) or PBS for 45 min and subcellular fractionation (nucleus vs. cytosol). Western blotting was conducted on the cytosolic fraction using antibodies against p-IκBα, IκBα, NF-κB, and β-actin (as a cytosolic marker). **(B–D)** Quantification of the data from **(A)** (p-IκBα: con, *n* = 12; LPS, *n* = 12; ALWPs + LPS, *n* = 12; IκBα: con, *n* = 12; LPS, *n* = 12; ALWPs + LPS, *n* = 12; NF-κB: con, *n* = 12; LPS, *n* = 12; ALWPs + LPS, *n* = 12). **(E)** Western blotting was performed on the nuclear fraction using antibodies against NF-κB and PCNA (as a nuclear marker). **(F)** Quantification of the data from **(E)** (con, *n* = 12; LPS, *n* = 12; ALWPs + LPS, *n* = 12). **(G)** BV2 microglial cells were pretreated with ALWPs (500 μg/ml) or PBS for 5 h and then treated with LPS (1 μg/ml) or PBS for 45 min. Cells were fixed and immunostained with anti-CD11b and anti-p-NF-κB (Ser536) antibodies (40× confocal images). **(H)** Quantification of the data from **(G)** (con, *n* = 122 cells; LPS, *n* = 129 cells; LPS + ALWPs, *n* = 111 cells). **(I)** BV2 microglial cells were pretreated with PF-573228 (a FAK inhibitor, 5 μM) or vehicle (1% DMSO) for 30 min, followed by treatment with ALWPs (500 μg/ml) or PBS for 30 min and finally LPS (1 μg/ml) or PBS for 5 h. Cells were fixed and immunostained with anti-CD11b and anti-p-NF-κB (Ser536) antibodies. **(J)** Quantification of the data from **(I)** (con, *n* = 260 cells; LPS, *n* = 331 cells; LPS + ALWPs, *n* = 265 cells; LPS + FAK inhibitor, *n* = 188 cells; LPS + FAK inhibitor + ALWPs, *n* = 199 cells). Scale bar 20 μm (40× confocal images). ^∗^*p* < 0.05, ^∗∗∗^*p* < 0.001.

NF-κB plays an important role in neuroinflammation (Lawrence, 2009; Cao et al., 2010; Tilstra et al., 2011; Yao et al., 2013a). NF-κB in microglia is activated by viruses and bacterial toxins such as LPS. For instance, the activation of NF-κB in patients with chronic inflammation has been found to be a critical factor for AD, PD, and osteoporosis, which are autoimmune/inflammatory diseases (Vallabhapurapu and Karin, 2009). Therefore, we examined whether ALWPs can regulate NF-κB subcellular localization. BV2 microglial cells were pretreated with ALWPs (500 μg/ml) or PBS for 5 h, treated with LPS (1 μg/ml) or PBS for 45 min, and then subjected to subcellular fractionation (nucleus vs. cytosol). We observed that pretreatment with ALWPs followed by LPS did not alter p-IκBα, IκBα, or NF-κB levels in the cytosol compared with LPS treatment (**Figures 6A–D**). In the nuclear fraction, LPS increased NF-κB levels compared with control treatment (**Figures 6E,F**). Pretreatment with ALWPs followed by LPS resulted in decreased LPS-induced nuclear NF-κB levels compared with treatment with LPS alone in BV2 microglial cells (**Figures 6E,F**).

To further confirm our findings, BV2 microglial cells were pretreated with ALWPs (500 μg/ml) or PBS for 5 h, followed by treatment with LPS (1 μg/ml) or PBS for 45 min. Immunocytochemistry was then performed with anti-CD11b and anti-p-NF-κB antibodies. LPS alone significantly increased nuclear p-NF-κB levels, whereas ALWPs significantly decreased LPS-induced nuclear p-NF-κB levels in BV2 microglial cells (**Figures 6G,H**). These data suggest that ALWPs modulate LPS-induced NF-κB nuclear translocation in BV2 microglial cells.

We then examined whether ALWPs modulate FAK signaling to alter LPS-induced nuclear p-NF-κB levels. BV2 microglial cells were pretreated with the FAK inhibitor PF-573228 (5 μM) for 30 min, followed by treatment with ALWPs (500 μg/ml) or PBS for 30 min and finally LPS (1 μg/ml) or PBS for 5 h. Immunocytochemistry was then conducted with anti-CD11b and anti-p-NF-κB antibodies. Treatment with PF-573228, ALWPs, and LPS further decreased LPS-stimulated nuclear p-NF-κB levels compared with treatment with PF-573228 and LPS or ALWPs and LPS (**Figures 6I,J**). These data suggest that ALWPs alter FAK signaling to affect nuclear p-NF-κB signaling to modulate the neuroinflammatory response.

### ALWPs Reduce LPS-Induced Microglial Cell Migration

**FIGURE 7:**
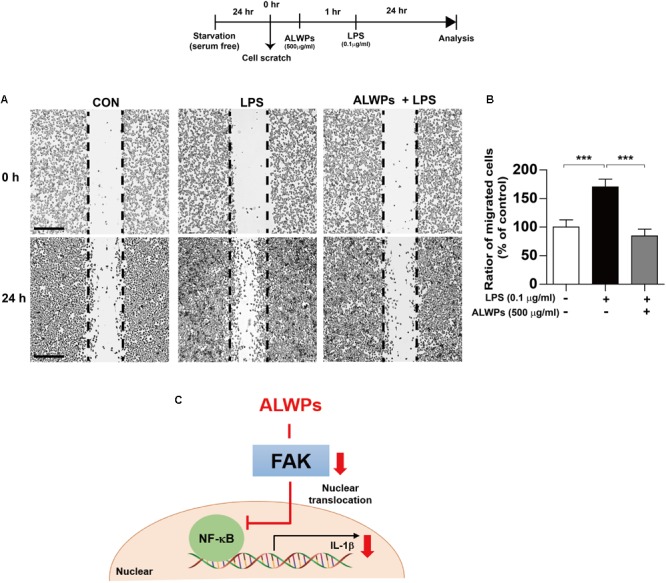
ALWPs inhibited LPS-induced BV2 microglial cell migration. **(A)** BV2 microglial cell monolayers were scratched with a fine tip, pretreated with PBS or ALWPs (500 μg/ml) for 1 h, and then treated with LPS (0.1 μg/ml) or PBS for 23 h. Images of the wound gap were acquired at 0 h (i.e., immediately after scratching) and after 24 h. Scale bar 200 μm (4× confocal images). **(B)** Quantification of the data from **(A)** (con, *n* = 16; LPS, *n* = 16; ALWPs + LPS, *n* = 16). **(C)** A working model of the regulation of the LPS-induced neuroinflammatory response by ALWPs. ^∗∗∗^*p* < 0.001.

Several studies have shown that microglial cell migration is associated with the neuroinflammatory response (Choi et al., 2010; Karlstetter et al., 2011; Bose et al., 2016). To examine whether ALWPs can regulate microglial cell migration, we conducted wound-healing assays in which BV2 microglial cells were pretreated with PBS or ALWPs (500 μg/ml) for 1 h and treated with LPS (0.1 μg/ml) or PBS for 24 h. We found that LPS alone significantly increased BV2 microglial cell migration compared with control treatment (**Figures 7A,B**). In addition, pretreatment with ALWPs followed by LPS treatment significantly suppressed BV2 microglial cell migration compared with LPS treatment (**Figures 7A,B**). These data suggest that ALWPs can inhibit LPS-mediated BV2 microglial cell migration.

### ALWPs Rescue Cognitive Performance and Inhibit Microglial Activation in LPS-Injected Wild-Type Mice

**FIGURE 8:**
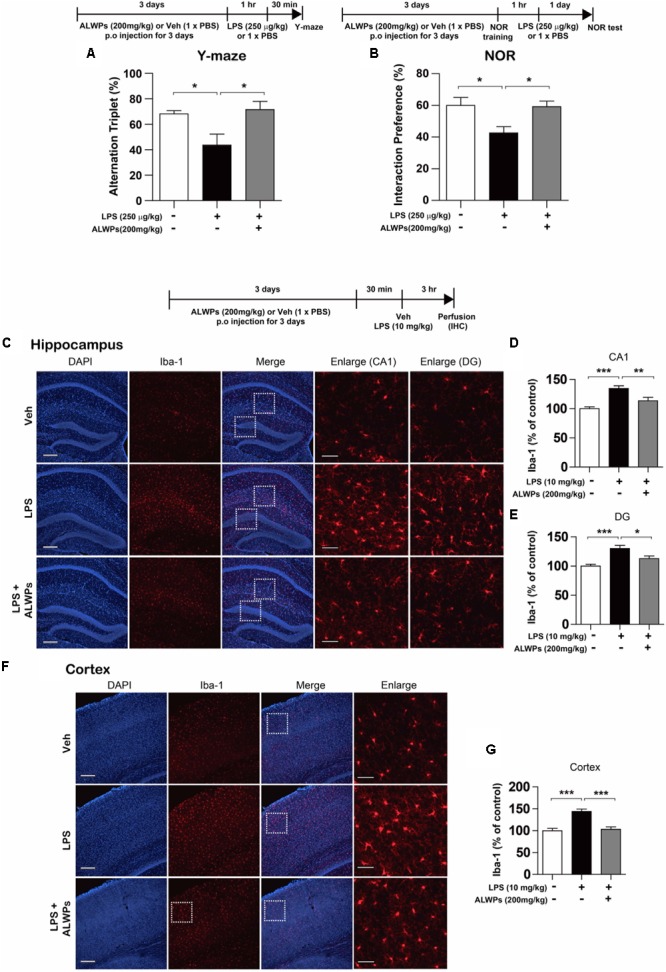
ALWPs rescued impaired short- and long-term memory in LPS-injected wild-type C57BL6/J mice. **(A,B)** Wild-type C57BL6/J mice were orally administered ALWPs (200 mg/kg) or PBS daily for 3 days. On the third day, 1 h after treatment with PBS or ALWPs, LPS (250 μg/kg) was injected intraperitoneally (i.p.). Y-maze tests and training sessions of the novel object recognition test (NOR) were performed 1 h after LPS injection. NOR tests were conducted 24 h after the NOR training session (Y-maze: Veh, *n* = 9/mice; LPS + Veh, *n* = 9/mice; LPS + ALWPs, *n* = 11/mice; NOR test: Veh, *n* = 6/mice; LPS + Veh, *n* = 10/mice; LPS + ALWPs, *n* = 8/mice). **(C–G)** Wild-type C57BL6/J mice were orally administered ALWPs (200 mg/kg, p.o.) or PBS daily for 3 days. On the third day, LPS (10 mg/kg) was injected i.p Three hours later, immunohistochemistry was performed with an anti-IbaI antibody in the hippocampus [**(C–E)**, CA1: Veh, *n* = 3/mice; LPS + Veh, *n* = 3/mice; LPS + ALWPs, *n* = 3/mice, DG: Veh, *n* = 3/mice; LPS + Veh, *n* = 3/mice; LPS + ALWPs, *n* = 3/mice] and cortex [**(F–G)**, Cortex: Veh, *n* = 3/mice; LPS + Veh, *n* = 3/mice; LPS + ALWPs, *n* = 3/mice]. Scale bar 200 μm (10× confocal images), Scale bar 50 μm (enlarged images), ^∗^*p* < 0.05, ^∗∗^*p* < 0.01, ^∗∗∗^*p* < 0.001.

Several studies have shown that microglial activation is a cause of AD and other neurological disorders (Wee Yong, 2010; Hirsch et al., 2012; Asai et al., 2015; Heneka et al., 2015). In addition, enhanced neuroinflammation can lead to memory impairment and eventually results in neurodegenerative diseases (Fang et al., 2010; Tweedie et al., 2012; Kempuraj et al., 2016). Thus, we investigated whether ALWPs can regulate cognitive function *in vivo*. Wild-type C57BL/6J mice were orally administered ALWPs (200 mg/kg) or PBS daily for 3 days. On the last day, after the treatment with PBS or ALWPs, LPS (250 μg/kg) was injected i.p., and Y-maze tests were performed. In addition, training sessions of the novel object recognition test (NOR) were performed 1 h after LPS injection, followed by the NOR test 24 h later. The LPS-injected wild-type mice exhibited significantly reduced short- and long-term memory compared with PBS-injected wild-type mice (**Figures 8A,B**). Pretreatment with ALWPs and injection with LPS rescued short- and long-term memory compared with LPS injection alone (**Figures 8A,B**), suggesting that ALWPs can affect learning and memory in LPS-injected wild-type mice.

Next, we examined whether ALWPs can regulate microglial activation *in vivo*. Wild-type C57BL/6J mice were orally administered ALWPs (200 mg/kg) or PBS daily for 3 days. On the last day, after the treatment with PBS or ALWPs, LPS (10 μg/kg) was injected i.p. Three hours later, immunohistochemistry was conducted with an anti-Iba-1 antibody, a marker for activated microglia. The LPS-injected wild-type mice exhibited significantly increased microglial activation in the hippocampus (**Figures 8C–E**) and cortex (**Figures 8F,G**) compared with PBS-injected wild-type mice. However, pretreatment with ALWPs significantly reduced microglial activation in the hippocampus (**Figures 8C–E**) and cortex (**Figures 8F,G**) in LPS-injected wild-type C57BL/6J mice. Taken together, these data suggest that ALWPs not only suppress microglial activation but also rescue impaired short-and long-term memory in LPS-injected wild-type C57BL/6J mice.

### Analysis of the Chemical Components of ALWPs by UHPLC

Four chemical standards corresponding to components of ALWPs were used to confirm the ALWP formula by UHPLC as described in the Materials and Methods: 5-hydroxymethyl-2-furaldehyde (5-HMF), morroniside, loganin and paeoniflorin. 5-HMF is one of the components of *R. glutinosa*, morroniside is present in *L. chinense*, loganin is present in *C. Officinalis* in, and paeoniflorin is present in *Moutan Cortex Radicis.* We observed that the retention times of 5-HMF, morroniside, loganin and paeoniflorin were approximately 4.059, 10.160, 11.494, and 12.019 min, respectively (**Supplementary Figure 4**). These results indicate that ALWPs contain the corresponding herbal ingredients.

To verify each herbal component of ALWPs by identifying the principal markers and to determine the content of the principal markers, UHPLC was performed. Calibration curves for 5-HMF, morroniside, and paeoniflorin were constructed with standard concentrations ranging from 10 to 100 μg/ml. The calibration curve of loganin was constructed with standard concentrations ranging from 10 to 1000 μg/ml. The regression equations and correlation coefficients (R^2^) of 5-HMF, morroniside, loganin, and paeoniflorin were as follows: *y* = 15200× – 8020, *R*^2^ = 0.999969; *y* = 1820× + 760, *R*^2^ = 0.999232; *y* = 397× – 1550, *R*^2^ = 0.999733; and *y* = 1910× – 455, *R*^2^ = 0.999335, respectively (**Supplementary Figure 5**). Using these equations, the quantities of 5-HMF, morroniside, loganin, and paeoniflorin in ALWPs were calculated (**Table 1**).

**Table 1:** The contents of 5-HMF, morroniside, loganin, and paeoniflorin in ALWPs.

Origin	Marker compound	RT (min)	UV wavelength	Amount (μg/mL)
*Rehmannia glutinosa*	5-HMF	4.059	238	0.534 ± 0.002
*Cornus officinalis*	Morroniside	10.160	240	1.493 ± 0.015
*C. officinalis*	Loganin	11.494	237	9.390 ± 0.289
*Moutan Cortex Radicis*	Paeoniflorin	12.019	231	1.222 ± 0.006

## Discussion

Many studies have shown that neuroinflammation is tightly associated with neurodegenerative diseases, including AD. However, very little research has focused on elucidating the molecular mechanisms underlying neuroinflammation. In this study, we examined the novel effects of ALWPs on cognitive performance and neuroinflammatory responses. Specifically, we found that ALWPs suppressed LPS-induced IL-1β levels in BV2 microglial cells but not in primary astrocytes. ALWPs also modulated TLR4/FAK signaling to decrease LPS-induced IL-1β levels. In addition, ALWPs regulated the nuclear localization of the transcription factor NF-κB, thereby alleviating neuroinflammatory responses. Moreover, we observed that oral administration of ALWPs to LPS-injected wild-type C57BL/6J mice rescued short- and long-term memory and reduced microglial activation.

Proinflammatory cytokines and neuroinflammation have been linked to neurodegenerative disease (Downen et al., 1999; Norden et al., 2016). IL-1 exists in two isoforms, IL-1a and IL-1β, and both are highly involved in neurodegenerative diseases (Shaftel et al., 2008). A recent study showed that chronic activation of LPS or IFN-γ regulates proinflammatory cytokines, including IL-1β, leading to neuronal dysfunction and neuronal death (Papageorgiou et al., 2016). Another study found that activated microglial cells release IL-1β, leading to dopaminergic neuronal death in a PD animal model (Chung et al., 2017). Other studies have demonstrated that the natural compound resveratrol inhibits LPS-induced IL-1β expression in a murine microglial cell line (N9 cells) and in primary microglial cells but not in primary astrocytes (Lu et al., 2010). Interestingly, we observed that ALWPs affected LPS-stimulated IL-1β levels in BV2 microglial cells (**Figure 2**), whereas no effect on LPS-induced changes in proinflammatory cytokine levels was observed in primary astrocytes (**Supplementary Figures 1, 2**). Our findings indicate that ALWPs specifically modulate LPS-induced proinflammatory cytokine IL-1β levels and have different effects depending on cell type. We speculate that ALWPs reduce IL-1β levels to regulate neuroinflammation as well as cognitive function. However, we have not fully addressed why ALWPs only regulate IL-1β levels in microglial cells. The effects of ALWPs on LPS-induced IL-1β levels will be investigated at the molecular level in future studies.

Lipopolysaccharide binds to TLR4 on the cell surface of microglial cells, which then release proinflammatory cytokines (Gaikwad et al., 2016). Therefore, TLR4 inhibitors or antagonists are candidates as therapeutic agents targeting neuroinflammation-related diseases. In the present study, ALWPs altered TLR4 signaling to modulate LPS-induced IL-1β levels (**Figure 4**). It is possible that ALWPs interfere with the association between LPS and TLR4 or between LPS and unknown receptors that interact with LPS because we observed that ALWPs decreased LPS-induced IL-1β levels in the absence of a TLR4 inhibitor. In addition, we observed that ALWPs significantly decreased cell-surface levels of TLR4 (**Figure 4**). Thus, ALWPs inhibit the interaction between LPS and TLR4 on the cell surface, thereby affecting neuroinflammatory responses. However, it is not clear whether ALWPs affect the interaction between LPS and unknown receptors to alter neuroinflammation. A future study will explore whether ALWPs can modulate the interaction between LPS and TLR4 or unknown receptors to regulate the LPS-induced neuroinflammatory response.

Lipopolysaccharide activates TLR4 and downstream signaling cascades such as MAP kinase signaling (EKR1/2, JNK and P38). MAPK signaling is a key factor for regulating proinflammatory cytokines in microglial cells. The expression of IL-1β is regulated by the MAPK signaling pathway in neuroglial cells, including microglia, astrocytes, and a microglial cell line (BV2) (Kaminska et al., 2009; Dang et al., 2014; Guo et al., 2016). Unexpectedly, we found that ALWPs did not alter the LPS-induced MAPK signaling pathway (**Supplementary Figure 3**). Thus, we examined another potential target, the FAK signaling pathway, which is a downstream signaling pathway activated by LPS (Wong et al., 2011). LPS treatment increases autophosphorylation of FAK (Tyr397) in murine macrophages and human synoviocytes. In addition, a study has shown that LPS-induced IL-6 levels are associated with FAK phosphorylation (Tyr397) (Zeisel et al., 2005). Thus, we examined the effects of ALWPs on FAK phosphorylation and observed that ALWPs decreased p-FAK (Tyr 397) in LPS-stimulated BV2 microglial cells (**Figure 5**). In addition, treatment with a FAK inhibitor, ALWPs, and LPS did not reduce LPS-induced IL-1β levels compared with treatment with a FAK inhibitor and LPS (**Figure 5**). These data suggest that ALWPs suppress FAK phosphorylation (Tyr 397) to regulate LPS-induced IL-1β levels.

NF-κB is a key transcription factor regulating inflammatory cytokines (Shaftel et al., 2008; Lawrence, 2009). Several studies have shown that LPS increases the phosphorylation of NF-κB-p65 *in vivo* and *in vitro* (Stylianou et al., 1992; Schutze et al., 1995; Bonaiuto et al., 1997; Dai et al., 2015). In addition, NF-κB binds to the IL-1β promoter region (Hiscott et al., 1993), and treatment with IL-1 agonists increases the phosphorylation of NF-κB (Malinin et al., 1997). Here, we observed that ALWPs downregulated p-NF-κB and translocation in the nucleus in LPS-stimulated BV2 microglial cells (**Figure 6**). In addition, ALWPs but not the individual components significantly decreased LPS-stimulated proinflammatory cytokine IL-1β levels (**Figure 3**), suggesting that ALWPs play an important role in regulating NF-κB and IL-1β levels and providing a new mechanism of the anti-inflammatory response.

Microglial cell migration is associated with neuroinflammation. Specifically, microglial migration and the related phagocytic activity can be activated by G-coupled receptors, which include chemokines (Guida et al., 2017). A recent study demonstrated that the chemokine CCL2 modulates microglial cell migration through the MEK/ERK and PI3K pathways (Bose et al., 2016). Karlstetter et al. found that curcumin, one of the major components of turmeric, has anti-inflammatory effects by inhibiting microglial cell migration (Choi et al., 2010; Karlstetter et al., 2011). Therefore, we examined the effects of ALWPs on microglial cell migration and found that ALWPs significantly inhibited LPS-induced BV2 microglial cell migration (**Figure 7**). Based on the literature and our findings, we speculate that ALWPs modulate BV2 microglial cell migration by altering LPS-mediated IL-1β levels. Thus, we will further investigate how ALWPs regulate BV2 microglial cell migration.

In this study, we examined the anti-inflammatory effects of ALWPs. ALWPs contain ten different herbs, and some of the components of ALWPs are known to be involved in inflammation. For instance, 5-HMF, one of the components of *R. glutinosa*, a component of ALWPs, inhibits reactive oxygen species (ROS) and NF-κB activity in TNF-α-induced vascular endothelial cells (Kim et al., 2013). *Corni fructus*, a component of ALWPs, has anti-inflammatory effects by suppressing COX-2 and iNOS levels through the downregulation of NF-κB binding activity in macrophages (Sung et al., 2009). Morroniside is a major component of *C. officinalis*, a component of ALWPs, and reduces proinflammatory cytokine IL-6 and IL-1β levels in a rat model of acute myocardial infarction (Yu et al., 2018). In addition, type 2 diabetic rats orally administered morroniside, a component of ALWPs, exhibit downregulated NF-κB activity in hepatic tissue (Park et al., 2009). Loganin is one of the components of *C. officinalis*, a component of ALWPs, and has been shown to suppress ApoCIII-induced proinflammatory cytokines and NF-κB phosphorylation in mouse adipocytes (Li et al., 2016). Thus, each component of ALWPs has antioxidant and anti-inflammatory effects in vascular endothelial cells, hepatic tissue, or adipose cells. However, whether the ten individual components of ALWPs have anti-inflammatory effects in LPS-induced BV2 microglial cells and in the brain is not clear. To test this, we examined the effects of each component of ALWPs on proinflammatory cytokine IL-1β levels and found that ALWPs but not the individual components decreased LPS-mediated IL-1β mRNA levels in BV2 microglial cells (**Figure 3**). Although each component of ALWPs is known to have a positive effect at the cellular level, we suggest that the combination of these ten ingredients in ALWPs has synergistic effects to reduce neuroinflammation.

Memory loss is one of the clinical symptoms of neurodegenerative diseases and is most commonly associated with AD. Memory deficits have been shown to be closely related to neuroinflammation. Specifically, activated microglia release proinflammatory cytokines that exacerbate memory loss in various diseases (Perry and Holmes, 2014; Lelios and Greter, 2018; Wendeln et al., 2018). In particular, several studies have demonstrated critical roles for IL-1β and TNF-α in the formation of learning and memory. For instance, increased levels of IL-1β are correlated with cognitive deficits in sepsis-associated encephalopathy and a repeated social defeat model (Imamura et al., 2011; McKim et al., 2016). Other studies reported that intrahippocampal injection or chronic overexpression of IL-1β in the hippocampus region leads to impairments of spatial memory (Gonzalez et al., 2009; Moore et al., 2009). Wang et al. reported that the proinflammatory cytokine IL-1β induces memory deficits by modulating the expression of GABA_A_ receptors through the P38 signaling pathway (Wang et al., 2012). Similarly, overexpression of TNF-α in neurons or glial cells impairs synaptic plasticity and learning and memory (Fiore et al., 2000). In addition, chronic LPS administration impairs learning and memory via TNF-α (Belarbi et al., 2012). These studies indicate that inflammation can affect cognitive function. Accordingly, several studies have evaluated the potential of traditional herbal medicines to inhibit neuroinflammation and ameliorate memory loss in inflammation-related diseases. For example, chrysophanol (a component extracted from *Rhubarb*), a traditional herbal medicine, attenuates memory deficits and neuronal death by inhibiting inflammation in diabetic mice (Chu et al., 2018). Anthocyanins, another herbal medicine, ameliorate hippocampus-dependent memory impairment and prevent neuroinflammation via the JNK/Akt/GSK3β axis in LPS-injected wild-type mice (Khan et al., 2018). In addition, Gu et al. (2016) have shown that paeoniflorin, a component of ALWPs, improves learning and memory and inhibits proinflammatory cytokine levels in a mouse model of AD. Another group reported that loganin, a component of ALWPs, decreases Aβ-induced inflammatory responses and ameliorates memory deficits induced by scopolamine (Hwang et al., 2017). However, there are few studies on the therapeutic effects and related mechanisms of each of the components of ALWPs in inflammation-related cognitive deficits. Based on the literature, we hypothesized that ALWPs affect cognitive performance by inhibiting neuroinflammation. To test this hypothesis, we examined the effects of ALWPs on cognitive function in LPS-injected wild-type mice. Interestingly, we found that ALWPs had anti-inflammatory effects (**Figures 2, 3**) and attenuated LPS-induced short- and long-term memory deficits in wild-type mice (**Figure 8**). Future studies will examine whether ALWPs regulate cognitive function through IL-1β as well as whether the individual components of ALWPs are sufficient to enhance learning and memory.

## Conclusion

In summary, this study showed that traditional herbal medicine ALWPs are a regulator of anti-inflammatory TLR4/FAK signaling cascades in LPS-induced BV2 microglial cells. In addition, treatment with ALWPs followed by LPS alters the subcellular localization of the transcriptional factor NF-κB. Moreover, ALWPs promote short- and long-term memory and inhibit microglial activation. Taken together, these data indicate that ALWPs might be a potential therapeutic anti-neuroinflammatory drug for neuroinflammation-related diseases, including AD.

## Author Contributions

Y-MW, JWK, and H-SH conceived and designed the study. J-YL, JN, WL, BJ, HN, H-JC, H-JK, YN, and YS acquired the data. J-YL, HN, JN, BJ, YN, and JK prepared the figures. YS, YJ, and Y-MW prepared the tables. J-YL, JN, JK, YN, YJ, Y-MW, JWK, YN, and H-SH wrote the manuscript.

## Conflict of Interest Statement

The authors declare that the research was conducted in the absence of any commercial or financial relationships that could be construed as a potential conflict of interest.
