# Supplementary material for: An Aqueous Extract of Herbal Medicine ALWPs Enhances Cognitive Performance and Inhibits LPS-Induced Neuroinflammation via FAK/NF-κB Signaling Pathways
Source: Front Aging Neurosci. 2018 Sep 26;10:269. doi: 10.3389/fnagi.2018.00269 (PMC6168635; doi:10.3389/fnagi.2018.00269)
Supplement: Supplementary file 1 [file Data_Sheet_1.docx]

**Supplementary Figures**

**An aqueous extract of herbal medicine ALWPs enhances cognitive performance and inhibits LPS-induced neuroinflammation via FAK/NF-κB signaling pathways**

Ju-Young Lee^1,6^, Bitna Joo^1,5,6^, Jin Han Nam^1^, Wonil Lee^1^, Hye Yeon Nam^1^ _,_ Yongtaek Seo^4^, Hye-Jin Kang^1^, Hyun-Ji Cho^1^, Young Pyo Jang^4^, Jeongyeon Kim^1^, Young-Man We^2,3,*^, Ja Wook Koo^1,5,^*, Hyang-Sook Hoe^1,*^

^1^Department of Neural Development and Disease, Korea Brain Research Institute (KBRI), 61, Cheomdan-ro, Dong-gu, Daegu, Korea. 41068; ^2^Oriental Medical Clinic Center, Hyoo Medical Clinic, Gangnam-gu, Seoul, Korea, 06134; ^3^College of Korean medicine, Wonkwang University, Iksandae-ro, Iksan, Jeonbuk, Korea, 54538; ^4^Division of Pharmacology, College of Pharmacy, Kyung Hee University, Hoegi-dong, Dongdaemun-gu, Seoul, Korea, 02447; ^5^Department of Brain & Cognitive Sciences, Daegu Gyeongbuk Institute of Science & Technology (DGIST), Daegu, Korea, 42988; ^6^These authors contributed equally to this work.

*Corresponding author

Hyang-Sook Hoe, Ph.D.: Department of Neural Development and Disease, Korea Brain Research Institute (KBRI), 61 Cheomdan-ro, Dong-gu, Daegu, Korea, 41068

E-mail: [sookhoe72@kbri.re.kr](mailto:sookhoe72@kbri.re.kr)

Ja Wook Koo, Ph.D.: Department of Neural Development and Disease, Korea Brain Research Institute (KBRI), 61 Cheomdan-ro, Dong-gu, Daegu, Korea, 41068

E-mail: [jawook.koo@kbri.re.kr](mailto:jawook.koo@kbri.re.kr)

Young-Man We, M.D., Ph.D.: Hyoo Medical Clinic Center, Teheran-ro, Gangnam-gu, Seoul, Korea, 06134

E-mail: [hyooclinic@naver.com](mailto:hyooclinic@naver.com)

**
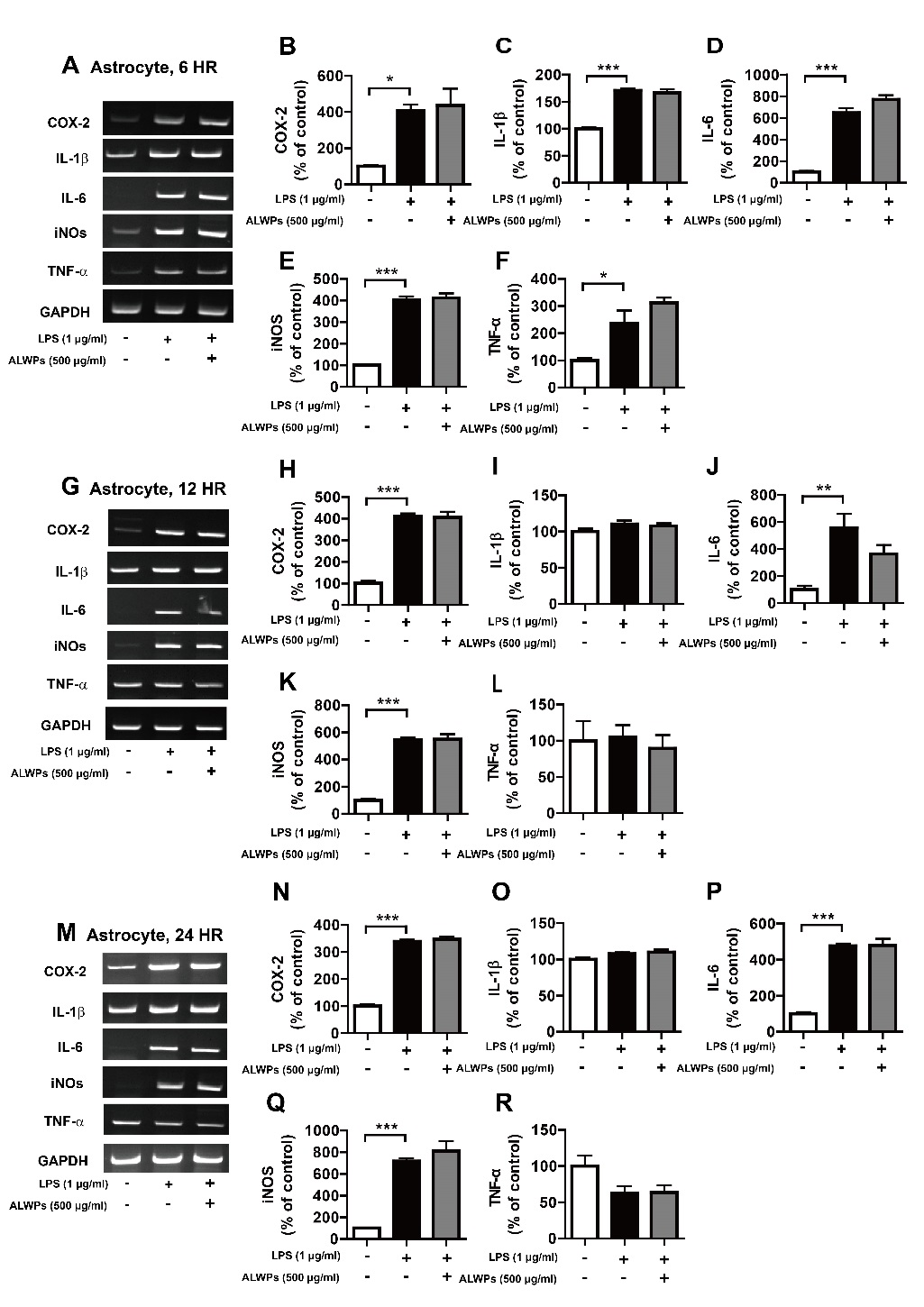
**

**Supplementary Figure 1**. ALWPs did not reduce LPS-stimulated pro-inflammatory cytokine levels at any time point in primary astrocytes. Primary astrocytes were pretreated with ALWPs (500 μg/ml) or PBS for 30 min, treated with LPS (1 μg/ml) or PBS for 5.5hr (A-F), 11.5 hr (G-L), or 23.5 hr (M-R). Total RNA was isolated, and the mRNA levels of pro-inflammatory cytokines were measured using RT-PCR (n=4/group). *p < 0.05, **p < 0.01, ***p < 0.001.

**
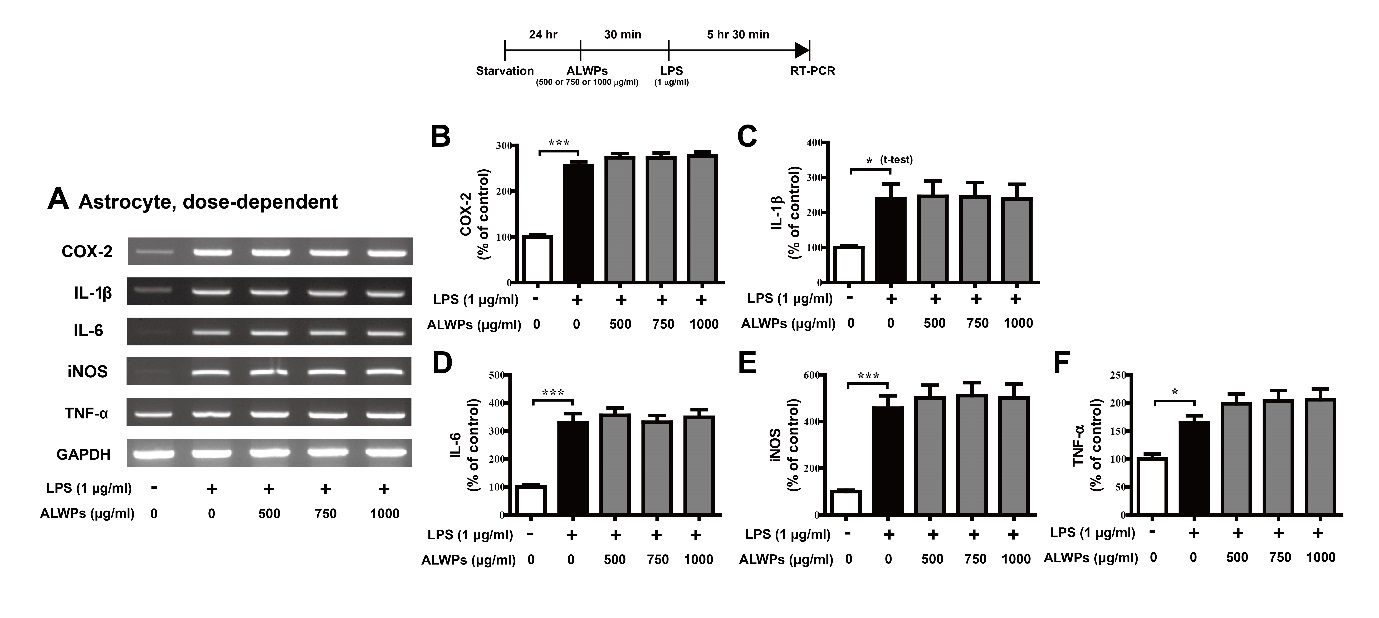
**

**Supplementary Figure 2**. No dosage of ALWPs did not alter LPS-induced pro-inflammatory cytokine levels in primary astrocytes. (**A-F**) Primary astrocytes were pretreated with ALWPs (500, 750, 1000 μg/ml) or PBS for 30 min, and treated with LPS (1 μg/ml) or PBS for 5.5 hr. Total RNA was isolated, and the mRNA levels of pro-inflammatory cytokines were measured using RT-PCR (n=8/group). *p < 0.05, ***p < 0.001.

**
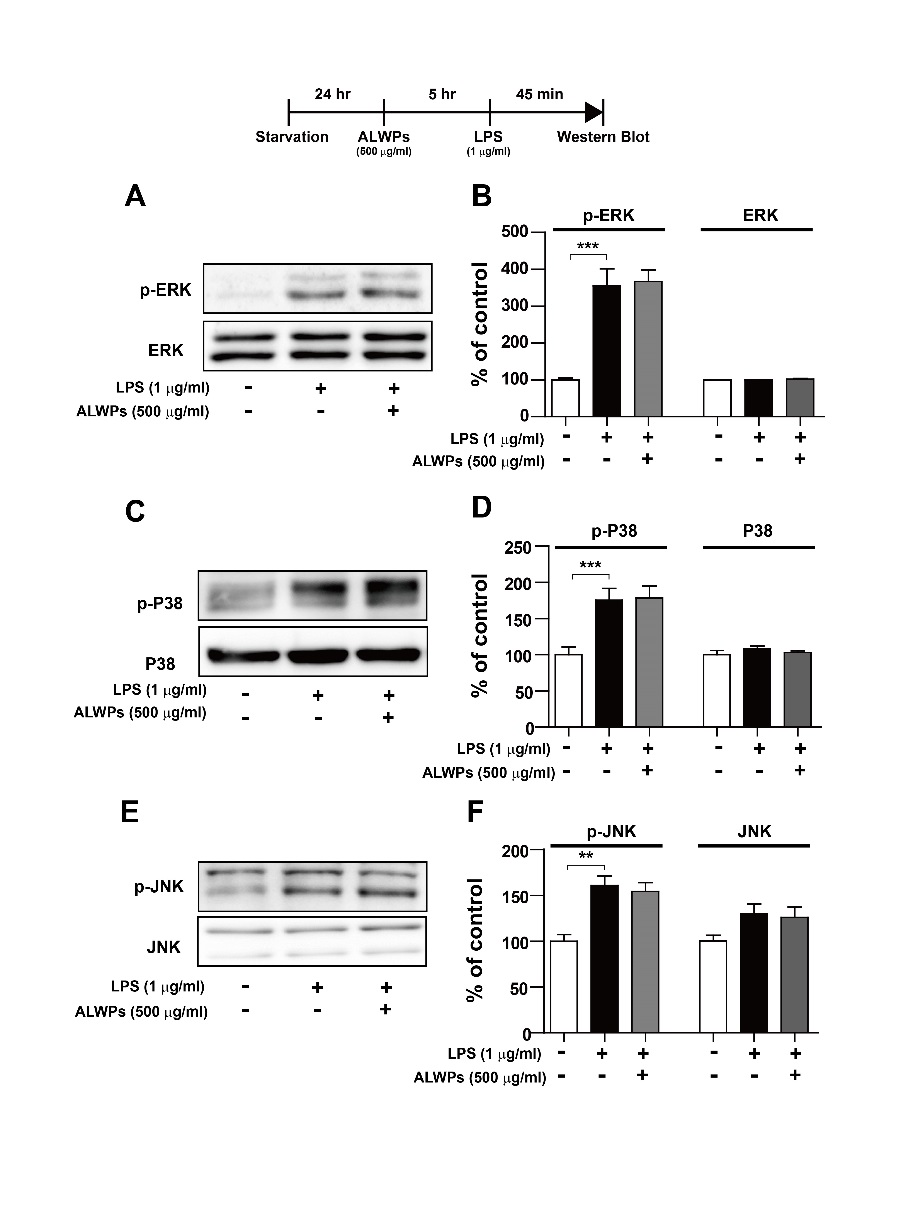
**

**Supplementary Figure 3**. ALWPs did not affect LPS-induced MAP kinase signaling in BV2 microglial cells. (**A**) BV2 microglial cells were pretreated with ALWPs (500 μg/ml) or PBS for 5 hr and then treated with LPS (1 μg/ml) or PBS for 45 min, and western blotting was performed with anti-p-ERK and anti-ERK antibodies. (**B**) Quantification of data from A (p-ERK: con, n=6; LPS, n=6; ALWPs + LPS, n=6, ERK: con, n=5; LPS, n=5; ALWPs + LPS, n=5). (**C**) BV2 microglial cells were pretreated with ALWPs (500 μg/ml) or PBS for 5 hr and then treated with LPS (1 μg/ml) or PBS for 45 min, and western blotting was performed with anti-p-P38 and anti-P38 antibodies. (**D**) Quantification of data from C (p-P38: con, n=6; LPS, n=6; ALWPs + LPS, n=6, P38: con, n=6; LPS, n=6; ALWPs + LPS, n=6). (**E**) BV2 microglial cells were pretreated with ALWPs (500 μg/ml) or PBS for 5 hr and then treated with LPS (1 μg/ml) or PBS for 45 min, and western blotting was performed with anti-p-JNK and anti-JNK antibodies. (**F**) Quantification of data from E (p-JNK: con, n=12; LPS, n=12; ALWPs + LPS, n=12; JNK: con, n=12; LPS, n=12; ALWPs + LPS, n=12). **p < 0.01, ***p < 0.001.

**
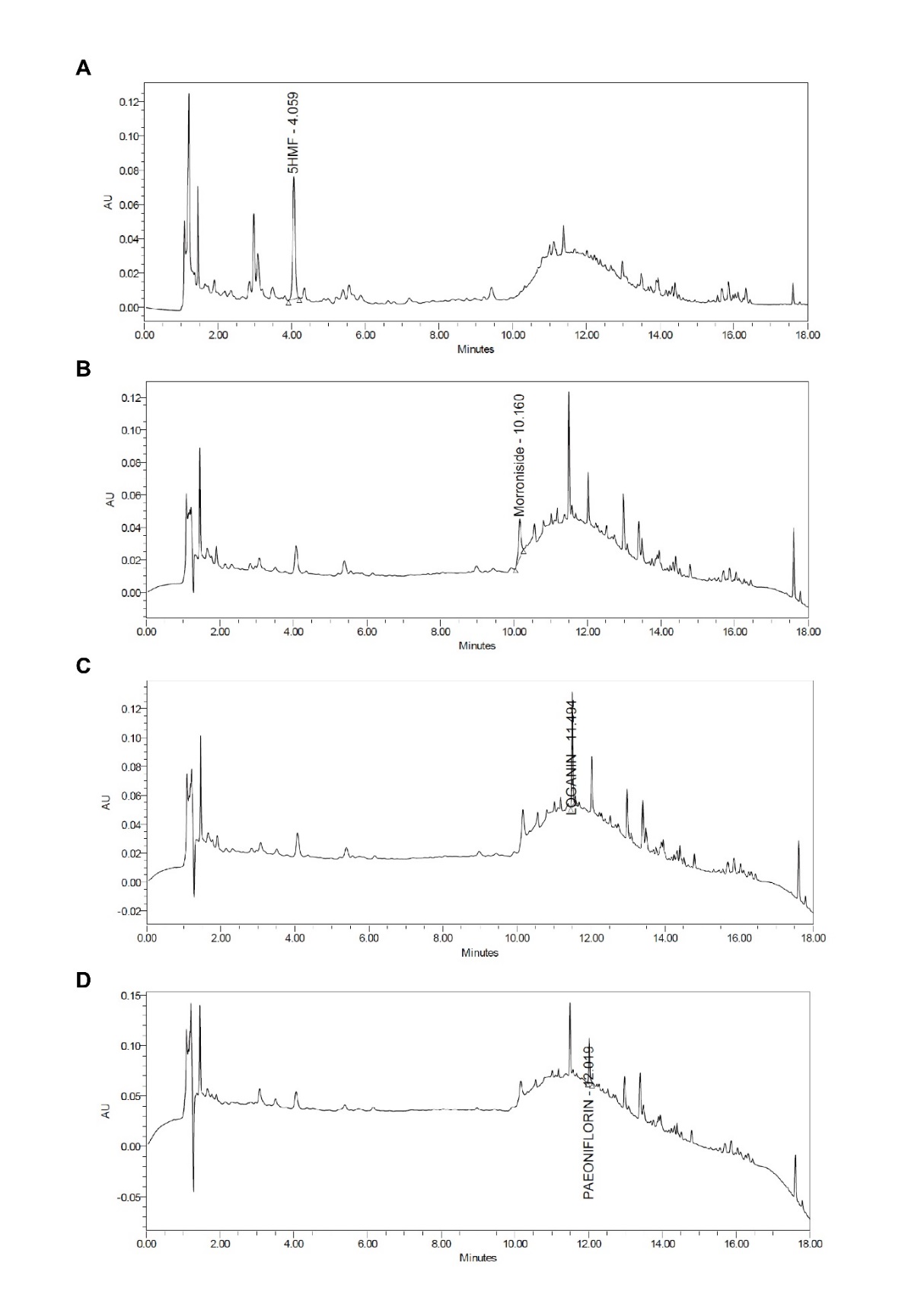
**

**Supplementary Figure 4.** Stacked plots of standards and wavelengths in ALWPs.

**
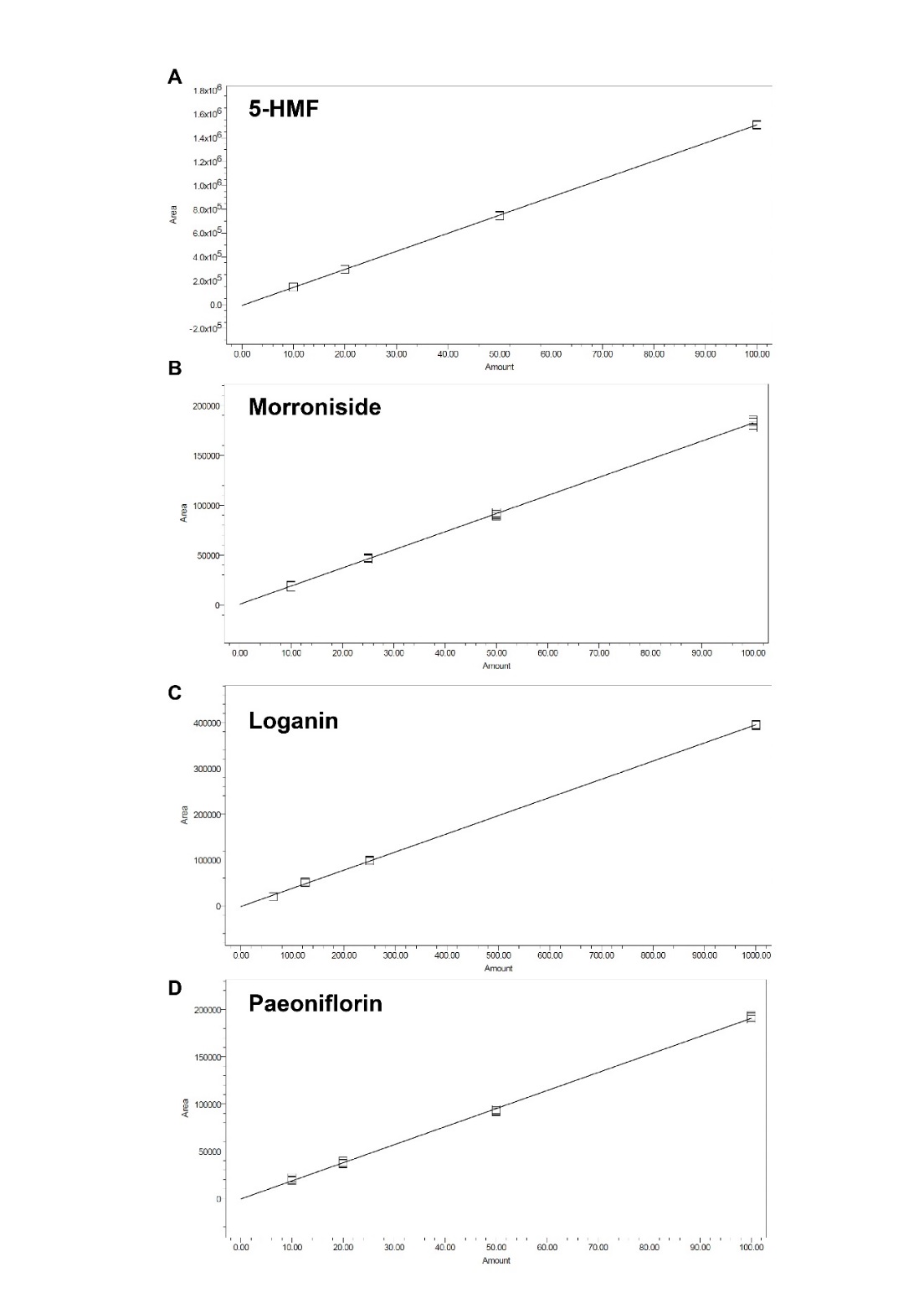
**

**Supplementary Figure 5.** Calibration curves of standards (5-HMF, morroniside, loganin, and paeoniflorin). (**A**) Calibration curve of standard 5-HMF, (**B**) Calibration curve of standard morroniside, (**C**) Calibration curve of standard loganin, (**D)** Calibration curve of standard paeoniflorin.
